# The Australasian COVID-19 Trial (ASCOT) to assess clinical outcomes in hospitalised patients with SARS-CoV-2 infection (COVID-19) treated with lopinavir/ritonavir and/or hydroxychloroquine compared to standard of care: A structured summary of a study protocol for a randomised controlled trial

**DOI:** 10.1186/s13063-020-04576-9

**Published:** 2020-07-14

**Authors:** Justin T. Denholm, Joshua Davis, David Paterson, Jason Roberts, Susan Morpeth, Thomas Snelling, Dominica Zentner, Megan Rees, Matthew O’Sullivan, David Price, Asha Bowen, Steven Y. C. Tong, for the ASCOT Investigator Group, Joshua Davis, Justin Denholm, Steven Tong, Megan Rees, David Paterson, David Price, Susan Morpeth, Matthew O’Sullivan, Jason Roberts, Thomas Snelling, Sue Anderson, Zoe McQuilton, Bala Venkatesh, Naomi Hammond, Vivekanend Jha, Victoria John Burston, James McMahon, Patrick Charles, Robert Commons, Daniel O’Brien, Andrew Mahoney, David Sheffield, Lyn-Li Lim, Brad Gardiner, Thomas Schulz, Joseph Torresi, Roy Chean, Joseph Sasadeusz, Ben Rogers, Craig Aboltins, Kasha Singh, Michelle Yong, David Lister, Kumar Visvanathan, James Molton, Alex Tai, Rachel Chalmers, Marianne Martinello, Paul Wilson, Timothy Gray, Sarah Coghill, Hong Foo, Archana Sud, Jacob Williams, Nilar Lwin, Jeffrey Post, Sebastian van Haal, Richard Sullivan, Gail Matthews, Benjamin Chi Hin Kwan, Andrew Slack, Omar Shum, Belinda Cochrane, Ravindra Dotel, Timothy Gray, Timothy Gilbey, Michael Mina, Yuen Su, Chris Trethewy, Bernie Hudson, Arunavo Chatterji, Christaan Mostert, David New, Edward Raby, Sionh Hui, Owen Robinson, Julie Hart, Shu Jin Tan, Astrid Arellano, Jonathan Chambers, Jane Davis, Nastaran Rafiei, Simon Smith, Marjoree Sehu, Janath da Silva, Paul Griffin, Andrew Henderson, Khin Chaw, Keat Choong, Andrew Burke, Christopher Heather, Sanjaya Senanayake, Mark Boyd, Emily Rowe, Mick Anagnostou, Ali Trad, Alison Ratcliff, Susan Morpeth, Jack Dummer, Hassan Bhally, Massimo Giola, Kate Grimwade, Catharina Li-Lin Chang, Ayesha Verrall, Stephen Hogg, Dalilah Restropo, Michael Maze, Stephen Ritchie, Craig Gedye, Judy Chang, Michael Maze, Priyanka Pillai, Katie Flanagan, Jocelyn Mora, Jenny Post, Eileen Lam, Roberta Littleford, Naomi Knoblauch, Sandy Slow

**Affiliations:** 1 Victorian Infectious Diseases Service, The Royal Melbourne Hospital, and Doherty Department University of Melbourne, at the Peter Doherty Institute for Infection and Immunity, 792 Elizabeth Street, Melbourne, Victoria Australia; 2 Menzies School of Health Research, Charles Darwin University, Darwin, Australia; 3 Department of Infectious Diseases, John Hunter Hospital, Newcastle, NSW Australia; 4 University of Queensland Centre for Clinical Research, Faculty of Medicine & Centre for Translational Anti-infective Pharmacodynamics, School of Pharmacy, The University of Queensland, Brisbane, Australia; 5 Department of Infectious Diseases, Royal Brisbane and Women’s Hospital, Brisbane, Queensland Australia; 6 Departments of Pharmacy and Intensive Care Medicine, Royal Brisbane and Women’s Hospital, Brisbane, Australia; 7 Division of Anaesthesiology Critical Care Emergency and Pain Medicine, Nîmes University Hospital, University of Montpellier, Nîmes, France; 8 Middlemore Hospital, Counties Manukau District Health Board, Auckland, New Zealand; 9 School of Public Health, University of Sydney, Sydney, New South Wales Australia; 10 Department of Cardiology, The Royal Melbourne Hospital and Department of Medicine, University of Melbourne, Melbourne, Victoria Australia; 11 Department of Respiratory Medicine, The Royal Melbourne Hospital and Department of Medicine, University of Melbourne, Melbourne, Victoria Australia; 12 Centre for Infectious Diseases and Microbiology, Westmead Hospital, Westmead, New South Wales Australia; 13 University of Sydney, Sydney, New South Wales Australia; 14 Centre for Epidemiology & Biostatistics, Melbourne School of Population & Global Health, The University of Melbourne, Melbourne, Victoria Australia; 15 Victorian Infectious Diseases Reference Laboratory Epidemiology Unit at the Peter Doherty Institute for Infection & Immunity, Royal Melbourne Hospital and The University of Melbourne, Melbourne, Victoria Australia; 16 Telehealth Kids Institute, Perth, West Australia Australia

**Keywords:** COVID-19, Randomised controlled trial, protocol, hydroxychloroquine, lopinavir, ritonavir

## Abstract

**Objectives:**

To determine if lopinavir/ritonavir +/- hydroxychloroquine will reduce the proportion of participants who survive without requiring ventilatory support, 15 days after enrolment, in adult participants with non-critically ill SARS-CoV-2 infection.

**Trial design:**

ASCOT is an investigator-initiated, multi-centre, open-label, randomised controlled trial. Participants will have been hospitalised with confirmed COVID-19, and will be randomised 1:1:1:1 to receive lopinavir /ritonavir, hydroxychloroquine, both or neither drug in addition to standard of care management.

**Participants:**

Participants will be recruited from >80 hospitals across Australia and New Zealand, representing metropolitan and regional centres in both public and private sectors. Admitted patients will be eligible if aged ≥ 18 years, have confirmed SARS-CoV-2 by nucleic acid testing in the past 12 days and are expected to remain an inpatient for at least 48 hours from the time of randomisation. Potentially eligible participants will be excluded if admitted to intensive care or requiring high level respiratory support, are currently receiving study drugs or their use is contraindicated due to allergy, drug interaction or comorbidities (including baseline QTc prolongation of 470ms for women or 480ms for men), or death is anticipated imminently.

**Intervention and comparator:**

Participants will be randomised 1:1:1:1 to:

*Group 1*: standard of care;

*Group 2*: lopinavir (400mg) / ritonavir (100mg) twice daily for 10 days in tablet form;

*Group 3*: hydroxychloroquine (800mg) 4x200mg administered 12 hours apart on Day 1, followed by 400mg twice a day for 6 days;

*Group 4*: lopinavir /ritonavir plus hydroxychloroquine.

**Main outcomes:**

Proportion of participants alive and not having required intensive respiratory support (invasive or non-invasive ventilation) at 15 days after enrolment. A range of clinical and virological secondary outcomes will also be evaluated.

**Randomisation:**

The randomisation schedule will be generated by an independent statistician. Randomisation will be stratified by site and will be in permuted blocks of variable block size. The randomised sequence allocation will only be accessible to the data management group, and site investigators will have individual participant allocation provided through a web-based trial enrolment platform.

**Blinding (masking):**

This is an open-label study, with researchers assessing the laboratory outcomes blinded to treatment allocation. No unblinding procedures relating to potential adverse effects are therefore required.

**Numbers to be randomised (sample size):**

We assumed that 5% of participants receiving standard of care would meet the primary outcome, aimed to evaluate whether interventions could lead to a relative risk of 0.5, assuming no interaction between intervention arms. This corresponds to a required sample size of 610 per arm, with a 5% two-sided significance level (alpha) and 80% power. The total sample size therefore is planned to be 2440.

**Trial Status:**

ASCOT protocol version 3, May 5, 2020. Recruitment opened April 4, 2020 and is ongoing, with planned completion of enrolment July 31, 2021.

**Trial registration:**

Australian New Zealand Clinical Trials Registry (ACTRN12620000445976). Prospectively registered April 6, 2020.

**Full protocol:**

The full protocol is attached as an additional file, accessible from the Trials website (Additional file 1). In the interest in expediting dissemination of this material, the familiar formatting has been eliminated; this Letter serves as a summary of the key elements of the full protocol.

## Supplementary information

**Additional file 1.**

## Data Availability

Data recorded for this trial will be stored securely in a dedicated research database, accessible only to authorised investigators and study monitors. Findings will be submitted for presentation at appropriate conferences and for publication in peer-reviewed literature. Study updates will also be made available through the ASCOT website (https://www.ascot-trial.edu.au/). Investigators will actively pursue opportunities for data sharing, including individual participant meta-analysis.

