# Supplementary material for: The Australasian COVID-19 Trial (ASCOT) to assess clinical outcomes in hospitalised patients with SARS-CoV-2 infection (COVID-19) treated with lopinavir/ritonavir and/or hydroxychloroquine compared to standard of care: A structured summary of a study protocol for a randomised controlled trial
Source: Trials. 2020 Jul 14;21:646. doi: 10.1186/s13063-020-04576-9 (PMC7359440; doi:10.1186/s13063-020-04576-9)
Supplement: Supplementary file 1 — Additional file 1. [file 13063_2020_4576_MOESM1_ESM.pdf]

Study Title	***Australasian COVID-19 Trial (ASCOT)***
**Abbreviated Title**	ASCOT
**Clinical trials registration**	ACTRN12620000445976
**Universal trial number**	U1111-1250-5165
**Protocol version/date**	Version 3 / dated May 18, 2020
**Protocol number**	ERM62646
**Funding source**	Role of funders in study design, analysis and decision to publish: None
**Study Sponsor**	University of Melbourne
**Co-ordination**	
**Co-ordinating centre**	The Peter Doherty Institute for Infection and Immunity (University of Melbourne)
**Collaborating bodies**	The Australasian Society for Infectious Diseases Clinical Research Network (ASID CRN)
**Investigators**	
**Co-ordinating Principal Investigators**	A/Prof Steven Tong[1,2,3,]<br>A/Prof Justin Denholm[1,2,4],<br>Professor Joshua Davis[3,5]
**Chief Investigators**	1. David Paterson[6]<br>2. David Price[2,4]<br>3. Matthew O'Sullivan[7]<br>4. James Molton[6,8]<br>5. Sanjaya Senanayake[9,10,11]<br>6. Andrew Burke[12]<br>7. Nicholas Anagnostou[13]<br>8. Owen Robinson[14,15]<br>9. Alison Ratcliff[16]<br>10. Jane Davies[1,17]<br>11. Susan Morpeth[18]<br>12. Michael Maze[19]<br>13. Lou Irving[3,4,20]<br>14. Jason Roberts[6]<br>15. Peter Wark[5,21]<br>16. Emily Rowe[22]<br>17. Sandy Hodge[23]

	18. Megan Rees[2]
**Coordinating and Chief Investigators' Affiliations**	1. Peter Doherty Institute for Infection and Immunity, Victoria, 3000, Australia<br>2. Royal Melbourne Hospital, Melbourne Health, Victoria, 3050, Australia<br>3. Menzies School of Health Research, Casuarina NT 0811<br>4. University of Melbourne, Victoria, 3010, Australia<br>5. Hunter New England Health, Lookout Road, New Lambton Heights, NSW, 2305<br>6. Centre for Clinical Research, Faculty of Medicine, The University of Queensland, Level 8, Building 71/918, UQCCR, RBWH Campus, Herston QLD 4029<br>7. Sydney Medical School, Centre for Infectious Diseases and Microbiology, NSW Health Pathology, Marie Bashir Institute for Infectious Diseases and Biosecurity, University of Sydney, Westmead Hospital, Level 3, ICPMR Building, Westmead NSW 2145<br>8. Western Health, VIC, Australia<br>9. The Canberra Hospital, Yamba Dr, Garran ACT 2605<br>10. Medical School, College of Health and Medicine, Australian National University, Florey Building 54 Mills Road, Acton ACT 2601<br>11. School of Public Health and Community Medicine, University of New South Wales, Sydney, NSW, 2052<br>12. The Prince Charles Hospital, 627 Rode Rd, Chermside QLD 4032<br>13. Flinders Medical Centre, Flinders Dr, Bedford Park SA 5042<br>14. Royal Perth Hospital, Victoria Square, Perth WA 6000<br>15. Fiona Stanley Hospital, 11 Robin Warren Dr, Murdoch WA 6150<br>16. Tasmanian Health Service, Department of Health and Human Services, HOBART, TAS 7001<br>17. Royal Darwin Hospital, 105 Rocklands Dr, Tiwi NT 0810<br>18. Middlemore Hospital, 100 Hospital Road, Otahuhu, Private Bag 93311, Auckland 1640<br>19. Centre for international Health, Department of Preventive and Social Medicine, Dunedin School of

	Medicine, University of Otago, PO Box 56, Dunedin 9054, New Zealand<br>20. Peter MacCallum Cancer Centre, 305 Grattan Street, Melbourne VIC 3000 Australia<br>21. University of Newcastle, University Dr, Callaghan NSW 2308<br>22. Royal Adelaide Hospital, Department of Infectious Diseases, Port Road, SA 5000<br>23. Adelaide Medical School, The University of Adelaide, SA 5005 Australia

Document history:

Version Number	Date	Summary of changes
1.0	23 March 2020	Final approved version submitted to HREC
2.0	1 April 2020	Updated post HREC review
3.0	18 May, 2020	Amendment to reflect developmental changes made in response to information gathered since the previous version

CONFIDENTIAL

This protocol is confidential and is the property of Doherty Institute, University of Melbourne. No part of it may be transmitted, reproduced, published, or used without prior written authorisation from Doherty Institute.

Statement of Compliance

This clinical trial will be conducted in compliance with all stipulation of this protocol, the conditions of the ethics committee approval, the NHMRC National Statement on ethical Conduct in Human Research (2007 and all updates), the Integrated Addendum to ICH E6 (R1): Guideline for Good Clinical Practice E6 (R2), dated 9 November 2016 annotated with TGA comments and the NHMRC guidance Safety monitoring and reporting in clinical trials involving therapeutic goods (EH59, 2016).

This clinical trial is not sponsored by any pharmaceutical company or other commercial entity.

Table of Contents

ASCOT STUDY SYNOPSIS 6

INVESTIGATOR AGREEMENT 9

CONTRIBUTORSHIP 10

1. INTRODUCTION 11

1.1 ABBREVIATIONS 11
1.2 BACKGROUND AND RATIONALE 12
1.3 OBJECTIVES AND HYPOTHESES 15
1.4 TRIAL DESIGN 15

2. METHODS 16

2.1 STUDY SETTING 16
2.2 ELIGIBILITY CRITERIA 16
2.2.1 Participant Inclusion criteria 16
2.2.2 Participant Exclusion criteria 17
2.3 TREATMENT OF STUDY PARTICIPANTS 18
2.3.1 Standard of care arm 19
Note: Multilobar infiltrates has been removed as this is typical for COVID-19 20
2.3.2 Active treatment factorial arm 20
2.3.3 Study drugs 21
2.3.4 Criteria for discontinuing or modifying allocated interventions 21
2.3.5 Strategies to improve adherence to protocol 21
2.4 OUTCOMES 22
2.4.1 Primary outcome 22
2.4.2 Secondary outcomes 22
2.4.3 Rationale for these outcome measures 23
2.5 TRIAL PROCEDURES 23
2.5.1 Participant timeline 23
2.5.2 Screening 26
2.5.3 Informed Consent 26
2.5.4 Randomisation and blinding 28
2.5.5 Study Visit Day Details 28
2.5.5.1 Day 0/Screening (-12 to 0 days) 28
2.5.5.2 Day 1 28
2.5.5.3 Day 2 29
2.5.5.4 Day 3 30
2.5.5.5 Days 4, 5, 6, 8, 9 30
2.5.5.6 Day 7 30
2.5.5.7 Day 10 31
2.5.5.8 Days 11-14 31
2.5.5.9 Day 15 31
2.5.5.10 Day 28 + 3 days 32
2.5.5.11 Day 90 + 14 days 32
2.5.6 Data and sample collection 32
2.5.7 Discontinuation/Withdrawal of participants from trial treatment 33
2.5.8 End of trial 34
2.6 STUDY TIMELINE 34
2.7 SAMPLE SIZE 34
2.7.1 Primary endpoint 34
2.8 ASSIGNMENT OF INTERVENTIONS 35
2.8.1 Allocation 35
2.8.2 Allocation concealment 36
2.8.3 Implementation 36
2.8.4 Blinding 36
2.9 DATA MANAGEMENT AND QUALITY ASSURANCE 36
2.9.1 Source data 36

2.9.2 Protocol Deviations and Serious Breaches *36*
2.9.3 Data Recording and Record Keeping *37*
2.9.4 Confidentiality *38*
2.10 STATISTICAL METHODS 38
2.10.1 Statistical analysis plan *38*
2.10.2 Interim analyses and stopping guidelines *39*
2.10.3 Provision for a Bayesian adaptive trial approach *40*
2.11 MONITORING AND TRIAL CO-ORDINATION 40
2.11.1 Trial co-ordination *40*
2.11.2 Data safety and monitoring board (DSMB) *40*
2.11.3 Study monitoring *41*
2.12 SAFETY 41
2.12.1 Adverse Reaction (AR) definition *41*
2.12.2 Reference Safety Information (RSI) definition *41*
2.12.3 Safety Critical Adverse Events *41*
2.12.4 Serious adverse events (SAEs) *42*
2.12.5 Suspected Unexpected Serious Adverse Reaction (SUSAR) definition *43*
2.12.6 Significant Safety Issue (SSI) *43*
2.12.7 Urgent Safety Measurement (USI) *43*
2.12.8 Causality *43*
2.12.9 Summary reporting of adverse events and adverse drug reactions *44*
2.13 ETHICAL CONSIDERATIONS 45
2.13.1 General ethical considerations *45*
2.13.2 Summary of potential harms and risks to study participants *46*
2.13.3 Informed consent *46*
2.13.4 Drug shortages *46*
2.14 REGULATORY APPROVALS 46
2.15 DATA HARMONISATION, ACCESS AND SHARING 46
2.15.1 Data harmonisation *46*
2.15.2 Data Access *47*
2.15.3 Data sharing *47*
2.16 DISSEMINATION POLICY 47

3. APPENDICES 48

3.1. TRIAL SITES 48
3.2. PLANS FOR BIOLOGICAL SPECIMENS 48
3.3. TRIAL GOVERNANCE 48
3.3.1. Trial Steering Committee *48*
3.3.2. Trial Management Committee *48*
3.3.3. Funding arrangements *48*
3.4. PREPARATION OF STUDY DRUG ORAL SOLUTION 49
3.4.1 Hydroxychloroquine *49*
3.4.2 Lopinavir / Ritonavir *49*

4. REFERENCES 51

ASCOT Study Synopsis

**TITLE**	Australasian COVID-19 Trial (ASCOT). A multi-centre randomised clinical trial to assess clinical, virological and immunological outcomes in patients with SARS-CoV-2 infection (COVID-19) treated with lopinavir/ritonavir and/or hydroxychloroquine compared to standard of care
**BACKGROUND**	There are no therapies known to be efficacious for SARS-CoV-2 infections. Several agents have shown laboratory activity and are entering clinical trials. Lopinavir/ritonavir (LPV/r) has demonstrated some laboratory activity and is available as an approved oral formulation for HIV. Hydroxychloroquine has demonstrated laboratory activity and is available as an approved oral formulation for malaria. Clinicians have reported using LPV/r in individual cases of SARS-CoV-2 infection, but demonstration of benefit or appropriate indications are unknown. LPV/r improved clinical outcomes in non-randomised studies for SARS-CoV infections and is included as a therapy in a current clinical trial for MERS-CoV infections.
**PRIMARY OUTCOME MEASURE**	Proportion of participants alive and not having required intensive respiratory support (invasive or non-invasive ventilation) at 15 days after enrolment.
**SECONDARY OUTCOME MEASURES**	<u>**Clinical**</u><br>1. WHO 7-point outcome scale (clinician assessed)<br>2. Mortality at 7, 15, 28, 90 days<br>3. Time to death (up to 90 days)<br>4. Length of hospital stay<br>5. Receipt of invasive or non-invasive ventilation in first 28 days<br>6. Length of receipt of invasive or non-invasive ventilation<br>7. Length of ICU stay<br>8. Presence of chest infiltrates on CXR or CT at day 3 and day 7<br>9. Time to defervescence from randomisation<br>10. Biomarker levels – CRP and LDH and D-dimer<br>11. Antibiotic use – number of days of use in first 10 days post randomisation<br>12. Safety. Any of the following adverse events in first 10 days. See section on adverse events for definitions.<br>12.1. Diarrhoea – grade 2 or greater<br>12.2. Nausea – grade 2 or greater<br>12.3. Vomiting – grade 2 or greater<br>12.4. Pancreatitis – grade 2 or greater<br>12.5. QTc prolongation (>500ms) 24 hours following initial dose of study drugs<br>13. Safety. Serious ventricular arrhythmia (including ventricular fibrillation) or sudden unexpected death in hospital

	14. Safety. Acute Kidney Injury (AKI) based on the modified Kidney Disease Improving Global Outcomes (KDIGO) criteria (increase in creatinine by >1.5 fold, or by >26.5micromol/L from baseline).<br><br>**Virologic**<br>Viral clearance. Proportion of patients with negative SARS-CoV-2 RT-PCR at day 3 and day 7 from upper or lower respiratory tract samples.
**STUDY DESIGN**<br><br>***Control group***<br><br>***Intervention groups***	Open label, multi-centre, randomized controlled trial (RCT)<br><br>Standard of care without specific antiviral therapy<br><br>Lopinavir (400mg) / ritonavir (100mg) (LPV/r) twice daily for 10 days<br>OR<br>Hydroxychloroquine 800mg twice a day for 1 day, followed by 400mg two times a day for 6 days<br>OR<br>LPV/r ***PLUS*** hydroxychloroquine in the above regimens
**STUDY DURATION**	April 2020 onwards
**NUMBER OF PARTICIPANTS**	Flexible (see sample size section for justification)
**INCLUSION CRITERIA**	1. Age ≥ 18 years<br>2. Confirmed SARS-CoV-2 by nucleic acid testing in the past 12 days<br>3. Able to be randomised within 12 days of symptom onset<br>4. Expected to be remain an inpatient for at least 48 hours from the time of randomisation
**EXCLUSION CRITERIA**	1. Currently admitted to an Intensive Care Unit (ICU) or a hospital area that is functioning as an ICU<br>2. Currently receiving acute intensive respiratory support (*invasive or non-invasive ventilation).* Note that participants already on non-invasive ventilation (either CPAP or BiPAP) in the community can still be recruited if they are continuing on their usual degree of NIV.<br>3. Currently taking LPV/r OR hydroxychloroquine<br>4. Known allergy or hypersensitivity to LPV/r OR hydroxychloroquine<br>5. Use of medications that are contraindicated with lopinavir/ritonavir OR hydroxychloroquine that cannot be replaced or stopped during the study period (see Table 1 and https://www.covid19-druginteractions.org/)<br>6. Currently on other investigational agents with targeted antiviral effects<br>7. Known cirrhosis or ALT or AST > 5x upper limit of normal<br>8. Previous participation in the trial<br>9. Known pregnancy<br>10. Known HIV infection not on antiretroviral therapy

	11. QTc ≥470ms for males, QTc ≥480ms for females<br>12. Treating team deems enrolment in the study is not in the best interest of the patient<br>13. Death is deemed to be imminent and inevitable within the next 24 hours
**RANDOMISATION**	Eligible participants will be randomized 1:1:1:1 in a factorial design. Randomization will be stratified by site and be in permuted blocks of variable size.
**BLINDING**	This will be an open-label study.
**SAMPLE SIZE CALCULATION**	At this stage in the epidemic it is not clear the likely numbers of patients that will present to study sites. To detect a reduction in the primary endpoint from 5% to 2.5% would require a sample size of 2,440. To detect a reduction in the primary endpoint from 10% to 5% would require a sample size of 1,224. To detect a reduction in the primary endpoint from 15% to 10% would require a sample size of 1,740.
**ANALYSIS**	Analysis of the primary outcome will be by modified intention to treat (all participants with data available for the primary endpoint will be analysed according to the treatment allocation, regardless of what treatment they received). A per protocol analysis will also be performed. The per protocol population is defined as 1) for the control group: did not receive any LPV/r or hydroxychloroquine; 2) for the intervention group: received at least 80% of possible doses of LPV/r AND/OR hydroxychloroquine; 3) has available data. We also plan pre-specified subgroup analyses for the following groups:<br>a) Aged >65 or ≤65 years<br>b) Participants receiving ACE inhibitor/ATII blocker therapy at the time of presentation<br>c) Receipt of study drug within 96 hours of fever onset<br>d) Receipt of study drug within 96 hours of first symptom onset<br>e) Mild disease vs moderate disease

Investigator Agreement

I have read the protocol entitled “Australasian COVID-19 Trial (ASCOT)”.

By signing this protocol, I agree to conduct the clinical trial, after approval by a Human Research Ethics Committee or Institutional Review Board (as appropriate), in accordance with the protocol, the principles of the Declaration of Helsinki and the good clinical practice guidelines adopted by the TGA [Integrated Addendum to ICH E6 (R1): Guideline for Good Clinical Practice E6 (R2), dated 9 November 2016 annotated with TGA comments].

Changes to the protocol will only be implemented after written approval is received from the Human Research Ethics Committee or Institutional Review Board (as appropriate), with the exception of medical emergencies.

I will ensure that trial staff fully understand and follow the protocol and evidence of their training is documented on the trial training log.

**Name**	**Role**	**Signature and date** *(dd-mmm-yyyy format)*

Contributorship

Name	Summary of contribution
A/Prof Steven Tong	- initiated study design, protocol development
A/Prof Justin Denholm	- initiated study design, protocol development
Prof Joshua Davies	- initiated study design, protocol development
Prof David Patterson	- protocol development, site investigator, Queensland lead
Dr David Price	- provided statistical expertise on the clinical trial design<br>- will conduct the primary statistical analysis
Matthew O'Sullivan	- protocol development, site investigator, NSW lead
James Molton	- protocol development, site investigator, Victoria lead
Sanjaya Senanayaka	- site investigator, ACT lead
Andrew Burke	- protocol development, site investigator
Nicholas Anagnostou	- protocol development, site investigator, South Australia lead
Owen Robinson	- protocol development, site investigator, Western Australia lead
Alison Ratcliff	- protocol development, site investigator, Tasmania lead
Jane Davies	- protocol development, site investigator, Northern Territory lead
Susan Morpeth	- protocol development, site investigator, New Zealand lead
Michael Maze	- site investigator, New Zealand lead
Lou Irving	- protocol development, respiratory lead
Jason Roberts	- protocol development, pharmacokinetic expertise and lead
Peter Wark	- protocol development, respiratory lead
Emily Rowe	- protocol development, site investigator
Megan Rees	- protocol development, respiratory lead
Sandra Hodge	- protocol development

1. Introduction

1.1 Abbreviations

ADR	Adverse drug reaction
AE	Adverse event
AKI	Acute Kidney Injury
ASID CRN	The Australian Society for Infectious Diseases Clinical Research Network
BIPAP	Bilevel positive airway pressure
CI	Chief Investigator – A researcher who contributes to the funding, planning, and running of the entire study
Co-I	Co-investigator (a clinician or research assistant who aids the PI at a site)
CPAP	Continuous positive airway pressure
CRP	C-reactive protein
CTN	Clinical trial notification (to the Therapeutic Goods Administration)
DOB	Date of Birth
DSMB	Data and safety monitoring board
EDC	Electronic data capture
ESKD	End-stage Kidney Disease
EUC	Electrolytes, urea & creatinine
eCRF	Electronic case report forms
FBC	Full blood count
GCP	Good clinical practice
GP	General practitioner
GWAS	Genome wide association study
HRN	Hospital record number
HREC	Human research ethics committee
ICH	International Conference on Harmonisation
ICU	Intensive care unit
ID	identification
ID physician	Infectious disease physician
IMP	Investigational Medical Product
KDIGO	Kidney Disease Improving Global Outcomes
LFT	Liver function test
NHMRC	National Health and Medical Research Council
NIV	Non-invasive ventilation
PI	Principal Investigator (a clinician responsible for one site)
RC	research co-ordinator (responsible for multiple sites)
RCT	Randomised control trial
SAE	Serious adverse event
SOP	Standard Operating Procedure
SUSAR	Suspected unexpected serious adverse reaction
TdP	torsade de pointes
TGA	Therapeutic Goods Administration

1.2 Background and Rationale

1.2.1 Overview

In December 2019 a novel coronavirus emerged from Wuhan China as the cause of a pneumonia syndrome. This SARS-CoV-2 is a betacoronavirus and related to SARS. At the time of writing (1/4/20) there were over 830,000 reported cases and >40,000 deaths globally with 4,559 cases and 19 deaths in Australia. The case fatality rate is still unknown but likely to be ~1%. By May 5 2020, there were >3.5 million cases and >250,000 deaths globally, with 6,847 cases and 96 deaths in Australia.

There are no known effective therapeutic options at this stage. Clinical trials have begun for many agents including lopinavir/ritonavir (LPV/r) and hydroxychloroquine. While clinical trials in China initially provided an opportunity to assess the impact on clinical outcomes, there are now few reported cases in China. Clinical trials in Australia and New Zealand will have value in: 1) contributing to understanding of clinical efficacy in local healthcare settings; 2) allowing detailed assessments of virological and immunological outcomes in a blinded manner; 3) providing therapeutic options to Australian and New Zealand patients in the context of a clinical trial.

1.2.2 Clinical spectrum of disease

SARS-CoV-2 causes a clinically significant and transmissible respiratory tract infection. In a large case series patients commonly had fever, cough and evidence of pneumonia on chest xray[1].

1.2.3 Therapeutic options

Several broad approaches to improving outcomes for severe viral infections can be considered, beyond optimal supportive care as appropriate. These approaches may include host immune modulation (for example, with the use of treatments such as steroids to reduce inflammation, or immune globulin to enhance specific responses), or interventions which aim to interfere with viral activity. Several antiviral medications have been previously investigated in clinical and laboratory studies for Sudden Acute Respiratory Syndrome (SARS) and Middle East Respiratory Syndrome (MERS), diseases caused by related coronaviruses (SARS-CoV and MERS CoV respectively). These have not established definite efficacy for these coronaviruses, but have suggested potential benefit from the use of LPV/r with or without interferon B[2-4]. Other antivirals, including ribavirin, appear to have been associated with increased mortality and are not considered suitable candidates for SARS-CoV-2.

As SARS-CoV-2 is a novel viral pathogen, little clinical trial data on antiviral therapy and impact on outcomes exists. Laboratory studies have demonstrated that several agents, including chloroquine and LPV/r, have activity in vitro against SARS-CoV-2[5]. Remdesivir, a novel nucleoside analogue, has been shown to have in vitro activity against a range of coronaviruses, but is not in clinical use[5,6]. One recently published study reported improvement in time to clinical improvement in the group receiving LPV/r, but no statistically significant improvement in mortality[7]. However, the study was substantially underpowered to detect an effect, and a large randomised trial is needed to establish the efficacy of LPV/r in hospitalised patients infected with SARS-CoV-2.

WHO have recommended that specific treatments for COVID-19 should not be occurring outside of clinical trials given the current lack of evidence and potential for harm of therapeutic agents.

1.2.4 Rationale for selection of therapeutic agents

As outlined above, LPV/r and chloroquine have both had *in vitro* demonstration of effectiveness against SARS-CoV-2 replication[5]. Both medications are currently available for use in Australia and New Zealand for other indications and have well-established safety profiles. Activities (EC50s) of hydroxychloroquine against the SAR-CoV-2 virus are in the low micromolar range[8] which represents the upper end of the free plasma concentration range encountered in clinical practice.

Extensive advice has been sought from those with experience in using chloroquine for malaria. Chloroquine and hydroxychloroquine have unusual pharmacokinetic properties. Absorption after oral administration is rapid and generally reliable but the total apparent volume of distribution is enormous (>100 L/kg) reflecting extensive tissue binding. The initial plasma or whole blood concentration profile in the treatment of acute illness is determined mainly by distribution processes and not by elimination. This is critical to understanding dosing and concentration profiles and associated risks with short course treatments. In the treatment of malaria (or COVID19) the initial (loading) doses are designed to "fill" the body so that concentrations that would take weeks to achieve without a loading dose, are achieved as soon as safely possible. To achieve blood levels of hydroxychloroquine above the in vitro IC50-value for SARS-CoV-2 (1.13 μM)[8], high loading doses of hydroxychloroquine are required. Simulated blood concentration time profiles of hydroxychloroquine using pharmacokinetic models suggest that two large loading doses of 800mg in the first day are required, followed by 400mg bd for subsequent days (Professor Nicholas White, personal communication). These doses are above the currently recommended doses from the Therapeutic Goods Administration in Australia for general use of hydroxychloroquine.

Key features of the safety profile of LPV/r

It is important to note that many of the reported adverse events (AEs) of LPV/r relate to longer term use and are very unlikely to be an issue with a 10-day course – this includes hyperlipidaemia, redistribution of body fat and hyperglycaemia. Furthermore, most data come from patients with HIV infection, who are unlikely to be representative of those being treated for COVID-19. Considering short term use, commonly reported adverse effects include diarrhoea and nausea. In the recent RCT among 199 COVID-19 patients[7], nausea was reported in 9.5% in the LPV/r group and 0% in the control group, vomiting in 6.3% versus 0%, and diarrhoea in 4.2% versus 2.1%. Diarrhoea was severe (grade 3 or 4) in no patients, and nausea/vomiting in only 1. In the product information, rare but serious AEs include hepatitis, pancreatitis, QT interval prolongation and PR interval prolongation – however it is noted that a definite causal relationship has not been established for any of these. In the Cao et al trial, none of these more serious AEs occurred at a higher frequency in the LPV/r arm than in the control arm, apart from prolonged QTc, which occurred in 1 patient (1.1%) in the LPV/r arm and none in the control arm[7].

Key features of the safety profile of hydroxychloroquine (HCQ)
Similar principles apply to HCQ. Many of the reported AEs relate to long term use. Retinopathy and neuromyopathy are both reported, but relate to the total cumulative dose, and monitoring for these is only recommended in those on long term therapy (meaning more than one year). Serious but rare AEs which could be relevant with shorter courses include prolonged QT interval (and resultant arrhythmias), hypoglycaemia (generally with concomitant diabetes treatments), and pancytopenia. Although there is little experience to date in the treatment of COVID19 the extensive previous experience in acute malaria suggests that serious toxicity (hypotension, arrhythmias, convulsions) should be unlikely at the doses being used. Common and less serious AEs include nausea and vomiting, vertigo, tinnitus and rash.

Chloroquine and hydroxychloroquine can block the inwardly rectifying Kir2.1 potassium (hERG) channel which delays ventricular repolarization and results in electrocardiographic QT prolongation. This is a risk factor for polymorphic ventricular tachycardia (TdP: torsade de pointes) but it is currently unclear whether the risk of TdP is actually increased with chloroquine or hydroxychloroquine.

An assessment of the tolerability of chloroquine (and by extrapolation likely tolerability of hydroxychloroquine at high dose) as used for uncomplicated malaria in the IMPROV study[9] has been provided to the ASCOT protocol writing group. Among patients treated with chloroquine at doses of 10mg/kg in the first day (either as a single dose or two divided doses) acute vomiting within 60 minutes occurred in 22/1,322 (1.7%), vomiting in the first 24 hours in 94/1355 (7%), nausea in the first 24 hours in 200/1346 (14.9%), and diarrhoea in the first 24 hours in 25/1352 (1.9%).

If there is significant gastrointestinal intolerance with the hydroxychloroquine 400mg bd maintenance dose, then the dose can be reduced to 200mg bd.

Implications of using LPV/r and HCQ in combination
The key AE of concern with combination use of these two drugs is prolonged QT interval and resultant arrhythmias. There are no clinical data on combining these two drugs. We have built in extra exclusion criteria (baseline prolonged (QTc), monitoring (ECGs) and stopping rules (prolonged QTc) because of this.

Additional monitoring for AEs
Due to the high doses of hydroxychloroquine and combination with LPV/r, there will be additional monitoring of specific AEs. Specifically:

- Assessment of gastrointestinal intolerance (nausea, vomiting, diarrhoea) within the first 4 hours of the initial drug administration.
- Recommendation to check and correct electrolyte abnormalities of K+ (aiming for ≥4.0 mmol/L) and Mg++ (aiming for ≥0.7 mmol/L)
- For the first 100 patients, ECGs will be obtained at baseline, 4 hr, 24 hr and day 4 after initial drug administration. Study drugs will be ceased if QTc >500ms or ΔQTc >60ms (compared to baseline). A cardiac electrophysiologist will independently review all baseline, 4 hr, 24 hr and day 4 ECGs in a blinded fashion for the first 100 patients to compare this ‘gold standard’ assessment with the automated ECG reading. The findings

from this initial analysis of QTc will be reported to the DSMB. If there are no concerning safety signals, the 4 hr ECG will not be required for the rest of the trial.
- Specific collection of data on the occurrence of cardiac arrhythmias until the time of hospital discharge or in-hospital death

1.2.5 Rationale for conducting a RCT in Australasia

It is expected that a substantial number of Australian and NZ residents may be infected with SARS-CoV-2, and based on international experience, a significant proportion will require hospitalisation (up to 20%) and be at risk for intensive care admission (up to 5%) and death (~1%). No established treatment to prevent these poor outcomes exists, and ASCOT will provide a controlled trial environment for two candidate medications to be used and evaluated.

The ASCOT study will allow for the efficacy and safety of these medications, alone and in combination, to be considered under closely monitored conditions.

Even if the study does not recruit sufficient numbers in itself for definitive conclusions for the primary endpoint of alive and not having required invasive or non-invasive ventilation at 15 days, it will contribute to broader global understanding and contribute to meta-analyses with other trials. It will also be important to determine the efficacy and safety of trial interventions within an Australasian context as the healthcare standards may differ from that of trials conducted elsewhere. Furthermore, detailed collection of samples will provide virological and immunological mechanistic insights into the efficacy or lack of efficacy of trial interventions.

An underlying principle is that we should provide randomised care rather than random care. At this stage, it is unknown whether the agents to be tested will provide benefit, harm or neither. There may be limited supply of these agents and until there is clear evidence of benefit or harm, the most judicious use is within the context of a clinical trial.

1.3 Objectives and hypotheses

We hypothesise that treatment with LPV/r +/- hydroxychloroquine will lead to improved clinical outcomes for hospitalised patients with SARS-CoV-2 infection.

Primary Objective: To determine if LPV/r +/- hydroxychloroquine will reduce the proportion of participants who survive without requiring invasive or non-invasive ventilation, 15 days after enrolment, in adult participants with non-critically ill SARS-CoV-2 infection.

1.4 Trial design

ASCOT is an investigator-initiated, multi-centre, open-label, randomised controlled trial. The study design will allow harmonisation with existing frameworks such as the Sentinel Travellers Research Preparedness Platform for Emerging Infectious Diseases (SETREP-ID, PI Thevarajan) and the Randomized, embedded, multifactorial adaptive platform trial for community-acquired pneumonia (REMAP-CAP) study (CI Steve Webb). Patients enrolled in ASCOT and who progress to requiring invasive or non-invasive ventilation can be enrolled in REMAP-CAP.

As it is too early to accurately predict the size and distribution of the SARS-CoV-2 epidemic the feasibility and sample size requirements of the study will be progressively considered. In

the initial design, consented participants will be randomised 1:1:1:1 on day 1 to receive either
i) standard of care without LPV/r or hydroxychloroquine,
ii) LPV/r;
iii) hydroxychloroquine, or
iv) LPV/r + hydroxychloroquine.

For patients that are clinically worsening at day 5 or beyond, the protocol allows for (but does not encourage) the treating clinician to commence LPV/r +/- hydroxychloroquine from day 5 onwards. Daily data will be collected for the first 15 days or until discharge, whichever is earlier. There will be a core dataset collected for all patients at all sites and enhanced and research data and biological samples for sites with capacity. Data will be harmonised with the ISARIC SARS-CoV-2 and REMAP-CAP protocols and CRFs (https://isaric.tghn.org/novel-coronavirus/). As long as the participant remains an inpatient, their medical records will be reviewed weekly until discharge or the 90-day time point, whichever occurs first.

Figure 1 – Overview of trial design

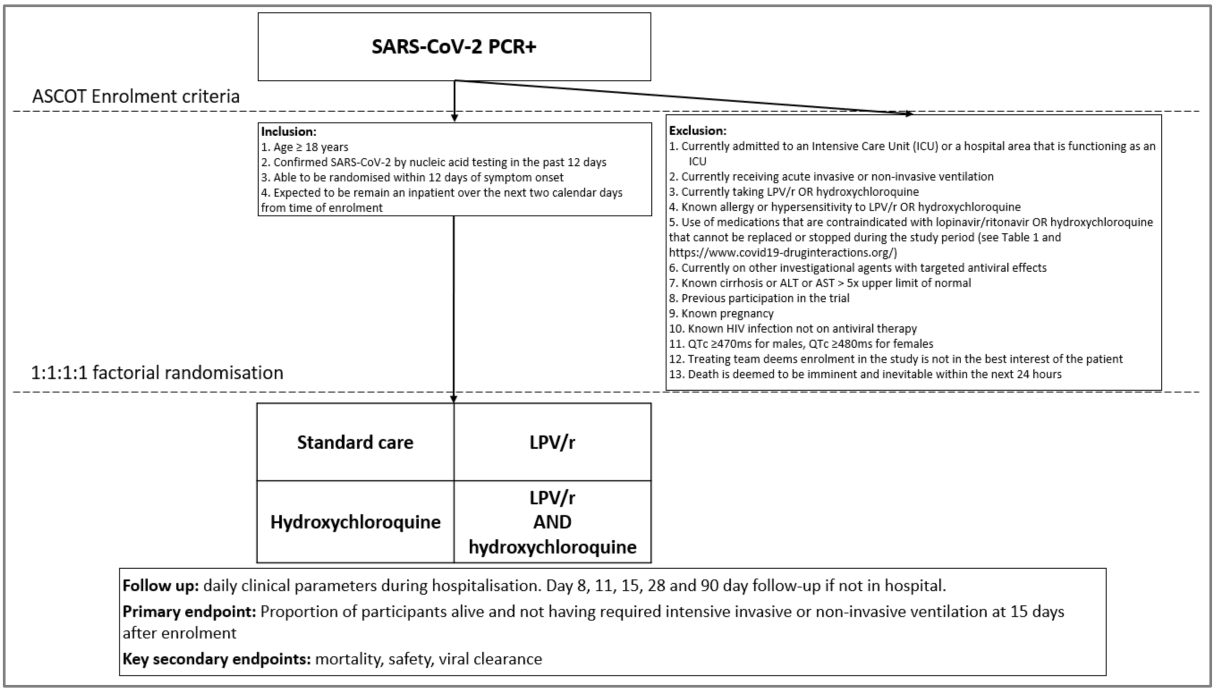

2. Methods

2.1 Study setting

We are aiming to recruit from sites across Australia and NZ. Sites will be selected on the basis of:
i) Estimated (or known) numbers of cases with a focus on larger sites;
ii) the availability of a committed principal site investigator and site research team; and
iii) capacity to collect samples as per the protocol.

2.2 Eligibility criteria

2.2.1 Participant Inclusion criteria

1. Age ≥ 18 years;
2. Confirmed SARS-CoV-2 by nucleic acid testing in the past 12 days;

3. Able to be randomised within 12 days of symptom onset;
4. Expected to be remain an inpatient for at least 48 hours from the time of randomisation.

2.2.2 Participant Exclusion criteria

1. Currently admitted to an Intensive Care Unit (ICU) or a hospital area that is functioning as an ICU;
2. Currently receiving acute intensive respiratory support (invasive or non-invasive ventilation). Note, participants already on non-invasive ventilation (either CPAP or BiPAP) in the community can still be recruited if they are continuing on their usual degree of NIV. Humidified high flow nasal oxygen will not be considered an exclusion criterion.
3. Currently taking LPV/r OR hydroxychloroquine
4. Known allergy or hypersensitivity to LPV/r OR hydroxychloroquine
5. Use of medications that are contraindicated with LPV/r OR hydroxychloroquine that cannot be replaced or stopped during the study period. (see table 1 and https://www.covid19-druginteractions.org/)
6. Currently on other investigational agents with targeted antiviral effects
7. Known cirrhosis or ALT or AST > 5x upper limit of normal
8. Previous participation in the trial
9. Known pregnancy
10. Known HIV infection not on antiretroviral therapy (see note below*)
11. QTc ≥470ms for males, QTc ≥480ms for females
12. Treating team deems enrolment in the study is not in the best interests of the patient
13. Death is deemed to be imminent and inevitable within the next 24 hours

Table 1: Drugs which should not be co-administered with study drugs

Drug Class	Drug Within Class Not to Be Co-administered
3A4 inhibitors	clarithromycin, erythromycin, diltiazem, itraconazole, ketoconazole, verapamil, fluconazole
3A4 inducers	phenobarbital, phenytoin, rifampicin
2D6 inhibitors	bupropion, fluoxetine, paroxetine, quinidine, metoclopramide
2D6 inducers	adalimumab, certolizumab, etanercept, golimumab, infliximab
QT prolongation	quetiapine, amiodarone, sotalol
HCV antivirals	glecaprevir/pibrentasvir

** For people with HIV on antiretroviral therapy significant drug-drug interactions may occur and potential changes in the antiretroviral regimen to facilitate enrolment in the trial should be done in conjunction with an experienced HIV prescriber. As guidance it is not recommended that individuals already receiving protease inhibitor, ritonavir, cobicistat, efavirenz, nevirapine or biktarvy should then be administered lopinavir/ritonavir. Antiretrovirals that are considered safe with lopinavir/ritonavir include tenofovir disoproxil fumarate (TDF) or tenofovir alefenamide (TAF), dolutegravir (DTG) and rilpivirine (RPV). It is recommended that individuals randomised to receive lopinavir/ritonavir should receive the lower dose formulation of tenofovir alefenamide if that forms part of their antiretroviral regimen.*

2.3 Treatment of Study Participants

Participants will be randomised to either the standard of care arm (2.3.1) or active treatment factorial (2.3.2). The standard of care arm will receive usual clinical care without LPV/r or hydroxychloroquine. The day of randomisation is considered day 1 of treatment. Randomisation and allocation must occur within 12 days of index sample collection that detected SARS-CoV-2 and within 12 days of symptom onset. The LPV/r or hydroxychloroquine will be prescribed by a doctor from the treating team as delegated. Storage conditions of LPV/r and hydroxychloroquine supplied by the Sponsor will be monitored for the study purposes. In the cases where institutional pharmacies have supplied LPV/r and/or hydroxychloroquine then the storage conditions will not be monitored. The participant's drug charts (electronic and/or paper) will be reviewed for compliance with study treatment. Any missed dose/s in the active treatment arms, and any use of LPV/r or hydroxychloroquine in the standard of care arm will be recorded on the CRFs.

Treating physicians are encouraged to keep to the duration of treatment as per the protocol. If treatment is prolonged past the protocol defined duration this will be recorded. For the purposes of analysis, the participant will be included in the allocated treatment arm as for intention to treat analysis. The participant will not be included in the per protocol analysis if ≥2 additional days of treatment have been given.

For participants who are enrolled into ASCOT AND who are taking an agent that is not on the contraindicated list (table 1) ***but*** that is potentially QT prolonging (table 2), clinical judgement is required. Consideration should be given to:

1. The relative importance of that medication;
    a. Could the medication be easily replaced with something else?
    b. Is there any harm in stopping the medication for 7-10 days?
2. Other patient-specific risk factors, such as;
    a. electrolyte disturbance, or presence of diarrhoea or use of diuretics that might predispose to electrolyte disturbance
    b. ischaemic heart disease or cardiomyopathy, history of ventricular arrhythmia
3. Recent use of QT prolonging agents with a long half-life, such as amiodarone
4. The number of potentially QT prolonging agents the patient is taking
5. The QTc interval on the patient's ECG; if it is <470ms for males and <480 for females while on the medication/s then it is not a requirement to stop the medication/s in question in order to prescribe the trial antiviral agents.

Note Azithromycin is not a contraindication to enrolment in ASCOT and use of hydroxychloroquine and/or lopinavir-ritonavir, but the considerations above should be thought through by the participant's clinical team.

Table 2: Example of Drugs which are known QT prolonging agents

Drug Name	
Amitriptyline	Use with caution
Azithromycin	Use with caution
Ciprofloxacin	Use with caution
Citalopram	Use with caution

Drug Name	
Clozapine	Use with caution
Domperidone	Contraindicated
Escitalopram	Use with caution
Flecainide	Contraindicated
Haloperidol	Use with caution
Lithium	Use with caution
Methadone	Use with caution
Mirtazapine	Use with caution
Moxifloxacin	Use with caution
Nortriptyline	Use with caution
Ondansetron	Use with caution
Propofol	Use with caution
Tacrolimus	Use with caution
Tamoxifen	Use with caution
Tramadol	Use with caution
Venlafaxine	Use with caution

For a more comprehensive list drugs which are QT prolonging agents

https://www.covid19-druginteractions.org/

2.3.1 Standard of care arm

Participants will receive usual medical care. The protocol does not allow the use of LPV/r or hydroxychloroquine in the first 5 days. If at day 5 the treating team decides to prescribe LPV/r or hydroxychloroquine, this will be permitted within the protocol and recorded. However, such action is not encouraged as:

1. There is no evidence to support either benefit or harm of antiviral agents in this setting;
2. The WHO has a clear statement that use of unproven therapeutic agents should occur in the context of a clinical trial;
3. In the setting of limited drug supplies, use should be prioritised to address clinical questions;
4. Study drug supplied by the Sponsor for the study will not be used in these cases. Sites will be required to acquire to LPV/r or hydroxychloroquine from their local institutional pharmacy through normal prescribing channels.

If LPV/r and / or hydroxychloroquine are used, the reasons for this decision will be recorded on the CRF and specifically will be:

1. Development of severe disease as per American Thoracic Guidelines for community acquired pneumonia as detailed in Table 3
2. Impending need for intensive respiratory support (invasive or non-invasive ventilation or humidified high flow nasal oxygen therapy)

Use of LPV/r or hydroxychloroquine outside of these reasons and if prior to day 5 will be considered protocol violations and a specific reason will be recorded.

Use of corticosteroids is discouraged[10] but recorded if used. Use of other potential therapies will also be discouraged but recorded if used. Antibiotic use will be recorded.

Table 3: American Thoracic Society criteria for severe community acquired pneumonia

Either one major criterion or three or more minor criteria
Minor criteria
Respiratory rate ≥ 30 breaths/min
PaO2/FiO2 ratio ≤ 250
New onset confusion/disorientation
Uremia (blood urea nitrogen level ≥ 7.14 mmol/L)
Leukopenia* (white blood cell count < 4,000 cells/µl)
Thrombocytopenia (platelet count < 100,000/µl)
Hypothermia (core temperature < 36°C)
Hypotension requiring aggressive fluid resuscitation
Major criteria
Septic shock with need for vasopressors
Respiratory failure requiring mechanical ventilation

Note: Multilobar infiltrates has been removed as this is typical for COVID-19

2.3.2 Active treatment factorial arm

Participants will be randomised 1:1:1:1 to:

Group 1: standard of care;

Group 2: lopinavir (400mg) / ritonavir (100mg) (2 tablets) twice daily for 10 days in tablet form;

Group 3: hydroxychloroquine 4x200mg (800mg) administered twice on Day 1, followed by 2x200mg (400mg) twice a day for 6 days; (maintenance dose can be reduced to 200mg bd if there is significant gastrointestinal intolerance).

Group 4: lopinavir / ritonavir plus hydroxychloroquine.

Hydroxychloroquine can be suspended and administered with the same dose and schedule as the tablet formulation. For participants who are unable to take medications by mouth, the LPV/r (400 lopinavir mg / 100 mg ritonavir) will be administered as a 5-ml suspension every 12 h via a pre-existing or newly placed nasogastric tube.. The Sponsor has been able to secure a limited stock of oral lopinavir/ritonavir solution. If during the study, the oral suspension is no longer available from the Sponsor, then the sites will be required to prescribe and administer study drug oral solutions as per the standard clinical pathways from their existing institutional stocks, otherwise refer to Appendix 3.4 for instructions on how to prepare oral suspensions.

The dose of LPV/r is the standard dosing used for treatment of HIV.. Because LPV/r oral solution contains ethanol and propylene glycol, it is not recommended for use with polyurethane feeding tubes due to potential incompatibility. Feeding tubes that are compatible with ethanol and propylene glycol, such as silicone and polyvinyl chloride (PVC) feeding tubes, can be used.

The initial dose of hydroxychloroquine is higher than the standard Australian recommendations (800mg/day max), which are for long term use. See section 1.2.4 for further details and rationale regarding the dosing of hydroxychloroquine.

The schedule for dosing of hydroxychloroquine will be 800 mg, administered twice on day 1 only. Subsequently, the dose will be 400 mg administered twice daily for 6 days. If there is

significant gastrointestinal intolerance with the hydroxychloroquine 400mg bd maintenance dose, then the dose can be reduced to 200mg bd.

For participants who have renal impairment, but not on dialysis, there will be no alteration in the hydroxychloroquine dosing schedule. Participants who are on dialysis (both haemodialysis or Peritoneal Dialysis) will have their dosing schedule changed to hydroxychloroquine 800mg twice on Day 1 followed by 400mg once daily for 6 days.

There is a risk of Torsades de Pointes with hydroxychloroquine and LPV/r and hence the study will exclude those with known QTC prolongation ≥470ms for males, QTc ≥480ms for females, and will monitor QTc during treatment. It is also recommended that electrolytes including Mg2+ should be checked at baseline and at day 2 and corrected if clinically indicated (aiming for K+ ≥ 4.0mmol/L and Mg2+ ≥ 0.70mmol/L. Consider discontinuing other medications that prolong QTc. See https://www.covid19-druginteractions.org (refer to table 2).

The duration of treatment is based on the natural history of worsening of clinical status at approximately one week of illness for patients who develop more severe disease. Treatment for 10 days with LPV/r will therefore extend past this critical time point. Blood levels of hydroxychloroquine will continue to be above the EC50 for SARS-CoV-2 at a similar time point given the long terminal half-life of hydroxychloroquine.

2.3.3 Study drugs

We have sourced study drugs from:
- Mylan for lopinavir/ritonavir (oral tablet);
- National Medical Stockpile (Australian Government) for Kaletra [lopinavir/ritonavir] (oral solution); and
- Sanofi for hydroxychloroquine.

These will be delivered to and stored at the Royal Brisbane and Women's Hospital (RBWH) pharmacy. RBWH pharmacy currently holds a cGMP license and has been accredited by the TGA as a COVID-19 central facility and will act as the central distribution warehouse for the ASCOT trial.

During the early phase of the study sites will be able to prescribe and administer study drugs as per the standard clinical pathways from their existing institutional stocks. All study sites have clinical pharmacies that provide clinical dispensing to hospitalised patients.

There will be a separate specific IMP management plan.

2.3.4 Criteria for discontinuing or modifying allocated interventions

2.3.4.1 Adjusting for renal function

Adjustment for renal function is not required for LPV/r or hydroxychloroquine.

2.3.5 Strategies to improve adherence to protocol

2.3.5.1 Training of site PIs

All site PIs will be trained in the study protocol, SOPs and their reporting requirements by the project manager, or a study chief investigator, prior to the site being opened for recruitment. All site PIs will need to have completed an accredited Good Clinical Practice training course.

The project manager will have regular phone contact with all enrolling site investigators, including after the enrolment of participants number 1, 2 and 5 at each site, and every 10 participants thereafter.

2.3.5.2 Documentation in patient's medical record and bedside chart

A sticker will be placed in the patient's medical record (one on the progress notes on the day of randomisation, and one in the front inside cover of the medical record ["old note"] if one exists). This sticker will alert clinicians that the patient has been randomised to the ASCOT study, with a brief explanation of the study.

A copy of the study synopsis will be placed in the bedside chart (observations and drug chart) of the patient. A checklist of study procedures will also be placed in the bedside chart.

For sites with electronic medical records and/or prescribing, an electronic "sticker" will be used, and appropriate annotations will be made to the electronic drug chart, or as per local institutional guidelines.

2.3.5.3 Checking of drug charts

The medication chart (be it paper or electronic) will be checked each day (apart from weekends) by the site PI or their delegate (registrar or research nurse) for the first 10 days whilst an inpatient to ensure adherence to the study protocol.

2.4 Outcomes

2.4.1 Primary outcome

Proportion of participants alive and not having new required intensive respiratory support (invasive or non-invasive ventilation) at 15 days after enrolment. This includes any participant who receives non-invasive mechanical ventilation (either CPAP or BIPAP) on the ward any time after enrolment even if not transferred to ICU. It does NOT include the use of humidified high-flow nasal prong oxygen. Participants on pre-existing home BiPAP or CPAP will not be considered to have met the primary endpoint unless they have either required invasive mechanical ventilation (i.e. intubation) or died by day 15.

2.4.2 Secondary outcomes

2.4.2.1 Clinical

1. WHO 7-point outcome scale (clinician assessed)
2. Mortality at 7, 15, 28, 90 days
3. Time to death
4. Length of hospital stay
5. Receipt of invasive or non-invasive ventilation in first 28 days
6. Length of receipt of invasive or non-invasive ventilation
7. Length of ICU stay
8. Presence of chest infiltrates on CXR or CT at day 3 and day 7
9. Time to defervescence from randomisation
10. Biomarker levels – CRP and LDH and D-dimer
11. Antibiotic use – number of days of use in first 10 days
12. Safety. Any of the following adverse events in first 10 days. See section on adverse events for definitions.

12.1 Diarrhoea – grade 2 or greater
12.2 Nausea – grade 2 or greater
12.3 Vomiting – grade 2 or greater
12.4 Pancreatitis – grade 2 or greater
12.5 QTc prolongation (>500ms) 24 hours following initial dose of study drugs

13. Safety. Serious ventricular arrhythmia (including ventricular fibrillation) or sudden unexpected death in hospital
14. Safety. Acute Kidney Injury (AKI) based on the modified Kidney Disease Improving Global Outcomes (KDIGO) criteria; serum creatinine increase by≥ 26.5µmol/L within 48 hours OR to ≥1.5 times baseline, known or presumed to have occurred within the prior 7 days

2.4.2.2 Virologic

15. Viral clearance. Proportion of patients with negative SARS-CoV-2 RT-PCR at day 3 and day 7 from upper or lower respiratory tract samples.

2.4.3 Rationale for these outcome measures

It is not clear at this stage how many patients will be suitable for enrolment. Therefore, there may not be sufficient patient numbers to power the study on clinical outcomes. If the case fatality rate is ~1%, the study will almost certainly not be powered for showing a difference in mortality. If the requirement for ventilation or mortality at 15 days is 5%, the study will require 2440 patients to demonstrate a difference of 2.5% in the primary endpoint. Nonetheless, the investigator group felt that the primary endpoint should be one of clinical significance. There is also value in conducting the study to assess virologic and immunologic outcomes. If viral clearance can be achieved more rapidly, in addition to potentially improving the clinical course, it would have implications for infection control and duration of hospitalisation.

The WHO Master Protocol is using a primary endpoint of an ordinal score at day 15:

1. Not hospitalized, no limitations on activities
2. Not hospitalized, limitation on activities;
3. Hospitalized, not requiring supplemental oxygen;
4. Hospitalized, requiring supplemental oxygen;
5. Hospitalized, on non-invasive ventilation or high flow oxygen devices;
6. Hospitalized, on invasive mechanical ventilation or ECMO;
7. Death.

To allow harmonisation with these WHO Master Protocol outcomes (https://www.who.int/blueprint/priority-diseases/key-action/COVID-19_Treatment_Trial_Design_Master_Protocol_synopsis_Final_18022020.pdf), the components for the ordinal scale will be part of the data collection. The ASCOT investigator group decided that analysis of these ordinal outcomes is complicated to evaluate any distinguishable/meaningful differences amongst these categories, and thus we have opted for a dichotomous primary endpoint.

2.5 Trial Procedures

2.5.1 Participant timeline

See Figure 1 and Table 4.

Table 4. Schedule of visits, data collection and follow-up.

Visit Day	Screening	On Treatment										Follow-up			EOS
	Day 0 (-12 to 0)	Day 1	Day 2	Day 3	Day 4	Day 5	Day 6	Day 7	Day 8	Day 9	Day 10	Days 11-14	Day 15	Day 28	Day 90
**CORE**															
Check eligibility	X														
ECG		X[5]	X[6]	✓	X	✓	✓	✓	✓	✓	✓	✓			
Blood glucose level (finger prick)			X[6]												
Informed consent		X													
Demographic data		X													
Clinical details		X													
Randomisation		X													
Notify GP		X													
Investigational drug dosing		X	X	X	X	X	X	X	X	X	X				
Investigational drug compliance check		X	X	X	X	X	X	X	X	X	X				
Clinical observations[1]		X	X	X	X	X	X	X	X	X	X	X	X	X	
Vital and ICU status[1]		X	X	X	X	X	X	X	X	X	X	X	X	X	
Patient Status															X
Routine chest imaging results[1, 2, 3, 4]		X	✓	X	✓	✓	✓	X	✓	✓	✓	✓	X		✓
Respiratory tract sample results[1]	X	X		X				X							
If discharged, contact participant via telephone/GP follow-up									X[9]			X[10]	X[11]	X	X
**Blood tests**															
FBC, EUC, LFTs, CRP[1,2]		X	X	X				X			X		X		
Lactate, D-dimer, coagulation studies[13], LDH, iron studies (including ferritin), troponin, BNP[1,3]		X		X				X			X		X		
K+ and Mg2+ levels[1,3]		X	X												
**PK testing (Optional)**															
Research bloods (1x3-6mL or 2x3-6mL)[1,8,12] plasma stored for PK analysis. Minimum 3mL required		X	X	(X)											

Visit Day	Screening	On Treatment										Follow-up			EOS
	Day 0 (-12 to 0)	Day 1	Day 2	Day 3	Day 4	Day 5	Day 6	Day 7	Day 8	Day 9	Day 10	Days 11-14	Day 15	Day 28	Day 90
**ENHANCED BIOLOGICAL (Optional Tier 1)**															
Research bloods[1] (1x9mL SST tube) for biobanking storage,		X		X				X					X	X	
Research bloods[1, 12] (1x5mL EDTA tube) for cell pellet (genomic) assay,		X													
Stool sample[1]		X													
Respiratory tract[1,7] sample stored	X							X							
**RESEARCH BIOLOGICAL (Optional Tier 2)**															
Research bloods (3x9mL Sodium Heparin and 2x9mL ACD tubes) for PBMC, plasma biobanking storage		X		X				X					X	X	

1. While still in hospital only
2. Part of routine care
3. ***Protocol suggested investigations, not mandatory. Local practices may recommend additional sample collection.***
4. Either CXR or CT chest. Initial CXR can be on day 1 or in the prior 24 hours.
5. ECG to be taken before 1st dose of study drug, and then 4 hours post.
6. ECG and blood glucose level to be performed 24 hours after the 1st dose of study drug (i.e. before day 2 dosing)
7. Clinical sampling for COVID-19 is a priority over research sampling. If routine clinical sampling swab is available for storage, then these will be stored, and test not repeated. If swabs are not available due to clinical shortage at the institution, then samples should not be collected.
8. Collect 1x EDTA for hydroxychloroquine arms <u>*and / or*</u> 1x lithium heparin blood tube for LPV/r arms. Refer to the laboratory manual for further information
9. Contact participant on Day 8 to enter data for Day 7
10. Contact participant on Day 11 to enter data for Day 10
11. Contact participant on Day 16 to enter data for Day 15
12. If site is participating in the PK and Enhanced Biological Optional tier 1 research sampling, then only 1x6mL EDTA tube is required to be collected. Otherwise refer to the laboratory manual
13. Coagulation studies not mandatory, but record any APTT, INR or fibrinogen results preformed as per local practice

✓ = if these tests performed at any other timepoint as part of usual COVID-19 care at site, results to be recorded in the CRF

2.5.2 Screening

All patients with a positive nucleic acid detection for SARS-CoV-2 will be referred by the pathology laboratory to the site investigator or their delegate (sub-investigator or properly qualified research nurse), as soon as identified. The following information will be transcribed onto a screening log by a member of the study team at the time of referral: date and time the sample was collected, the hospital record number (HRN), name & date of birth (DOB) of the patient and date and time the referral was received. The site investigator or their delegate will approach the doctors of the treating team and ask permission to approach the patient for potential recruitment onto the study and record their response in the screening log. To determine eligibility a screening paper CRF (CRF1) will be completed for all potentially eligible participants. The site investigator will do this using information gathered from the medical record and the patient's treating clinician. If the patient is eligible, the investigator will document this in the medical records. Following completion of CRF1, only patients who are eligible will be approached for an informed consent discussion. For ineligible patients and for patients who decline to participate, data may be able to be collected for observational studies. No identifiable data captured on CRF1 will leave the recruiting site.

2.5.3 Informed Consent

Due to the stringent measures in infection control in hospitals, verbal consent will be obtained instead of written consent. All patients will be in strict contact and droplet precautions and there will be an imperative to minimise use of personal protective equipment (PPE) by staff due to resource limitations. This has implications for recording of consent:

- Bringing a consent form and pen to the bedside and then taking these out of the room will violate the infection control rules.
- To minimise PPE use, only one person should enter a room to discuss the study with the patient. Therefore, a witness will not be present, neither will a next of kin be present. However, if an interpreter is required for the consent discussion, both the investigator and interpreter may enter the room together.
- It is acceptable to take the Patient Information Sheet and Consent form (PICF) into the room for viewing by the patient, and for these documents to be left in the room.
- Taking a photo of the signed consent form using site investigator staff digital phones was considered, but digital devices should not be used (outside of clinical requirements) with PPE.

An informed consent discussion will be held with each participant by a site investigator or their delegate. Study information that has been approved by the HREC will be provided in written format and in a language comprehensible to the potential participant, using interpreters if necessary. The participant will be given time to ask questions and consider whether to participate in the research. The PICF will remain in the patient's room, in accordance with infection control rules. The investigator will confirm the patient's agreement to the information detailed in the consent form by signing a separate copy of the form kept outside the isolation room. If phone facilities are available, visual contact can be made with the patient, and the PICF can be provided to the patient in written form, verbal consent may be obtained without entering the room.

After allowing the potential participant time to read the PICF, the investigator will answer any

additional questions they may have and will obtain verbal agreement to participate in the research. The person must clearly and orally indicate that they consent to participation in the study. The verbal consent will be recorded in both the participant's medical record and study consent form after the site investigator or delegate has left the room. This will state that the terms and conditions were all read and agreed to and all questions asked were answered. The following day, the investigator or clinical team will verbally confirm with the participant that they have consented to participate. A copy of the signed consent form will then be given to the participant either at discharge, via email or sent via post.

If a form is not available in a person's own language, the form must be translated verbally by an interpreter. The interpreter should sign the consent form held outside the isolation room that such a verbal and literal translation has been given. Where an interpreter is required, the interpreter will need to use appropriate PPE and follow local hospital policy procedures.

Prior to proceeding with randomisation, the investigator who has conducted the consent discussion will document in the medical records a summary of the discussion that includes: a statement that consent was obtained, details of any questions asked and answered and if applicable, that an interpreter was involved.

2.5.3.1 Surrogate Informed Consent

If the inclusion/exclusion criteria are confirmed, and the potential participant is unable to provide consent, then the person responsible/medical treatment decision maker (from this point referred to as person responsible) will be approached. At this time, the most senior member of the research team (investigator) will assess the competency of the person responsible before approaching them and inviting them to consider enrolling the potential patient into the ASCOT trial. If agreeable, the person responsible will be provided with further information about the study, both verbally and in written format (PICF). This will include a description of:

- ❖ The purpose of the study,
- ❖ Study procedures,
- ❖ Possible risks/benefits
- ❖ Rights and responsibilities of the potential participant, and
- ❖ Alternatives to participation.

The person responsible will be invited to ask questions which will be answered by the investigator, and they will be provided with contact details if they have any further questions. If the person responsible agrees that the potential patient should participate in the study, they will be asked to sign the informed Consent Form. A copy of the form will be given to them to keep. If the person responsible is indecisive about enrolment, they will be given 24 hours to consider the study and will be approached once more the following day.

The ASCOT managing trial centre recognises that different state jurisdictions have different requirements regarding surrogate consenting procedures. It is up to each site to consider whether they will submit the person responsible/medical treatment decision maker PICF to their HREC/RGO office, and to ensure jurisdictional approval has been received.

2.5.4 Randomisation and blinding

To randomise the participant the site investigator or their delegate will log onto the ASCOT database and enter the deidentified details from CRF1, which are required before randomisation and treatment assignment for that participant. Minimal compulsory fields required prior to randomisation are screening number, confirmation of eligibility, age, confirmation of consent.

Participants will be randomised in a 1:1:1:1 ratio to the standard of care or treatment arms, where the randomisation schedule will be generated by an independent statistician. Randomisation will be stratified by site and will be in permuted blocks of variable block size. The randomised sequence allocation will only be accessible to the data management group and as outlined in the data management SOP. As this is an open-label study and in the event of medical emergency no unblinding procedures are necessary.

2.5.5 Study Visit Day Details

2.5.5.1 Day 0/Screening (-12 to 0 days)

Screening activity, to evaluate eligibility will be undertaken as described in section 2.5.2 and will be undertaken whilst the patient is in hospital.

It is possible that Day 0 and Day 1 activities may be undertaken on the same day.

The activities undertaken at this visit are:

- Review of inclusion/exclusion and determine eligibility;
- Confirmation of a positive SARS-CoV-2 test within the last 12 days;

2.5.5.2 Day 1

These activities will occur on the day of randomisation and initiating study therapy.

Informed consent must be obtained before any study-specific screening evaluations are performed and should be documented in the participant's medical history (2.5.3.).

Any activities that are listed below and which were taken as part of routine care within 2 days of randomisation can be used and does not need to be repeated specifically for this study. Most information will be available from the Hospital Medical History. The evaluations/results that will be performed/recorded:

- Verbal informed consent;
- Demographic information (date of birth, age, sex, ethnicity);
- Chest imaging. A CXR or CT chest is not mandated investigation, and data will only be collected if this has been performed as part of routine care on day 1 or results from the preceding 24-hour period;
- Medical history including symptoms, comorbidities, BCG vaccination;
- Travel history (if applicable) in the previous 14 days;
- Review medications history (including any over-the-counter medications);
- Vital signs (blood pressure, heart rate, temperature);
- Review of routine clinical blood test results including full blood examination (FBE), biochemistry (i.e. EUC, LFTs, CRP);
- Clinical blood test, not mandated but recommended including, K+, Mg2+, lactate, D-dimer, LDH, coagulation studies (APTT, INR, fibrinogen) troponin, BNP. *If K+ or Mg2+*

are abnormal, then they should be corrected;

- 12 lead ECG;

Once eligibility has been confirmed the participant will be randomized. Prior to first dosing the following study specific activities will be undertaken:

- GP will be notified;
- *For sites involved in the enhanced biological component (tier 1):*
  - Research blood sample (1x9mL SST and 1x5mL EDTA blood tubes);
  - Stool sample;
  - Availability of respiratory tract samples from routine clinical care for biobanking from Day 0 and Day 1 OR Study specific nasopharyngeal and throat swab for biobanking
- *For sites involved in the research biological component (tier 2):*
  - Research blood samples (3x9mL sodium heparin tubes and 2x9mL ACD tubes);

Initial study drug will be administered. Record the time.

4 hours post first dose:

- 12 lead ECG at 4 hours post initial dose (this requirement may be removed depending on review after the first 100 participants have been assessed);
- *For sites involved in the PK component:* Research blood sample (1x5mL EDTA for hydroxychloroquine arms and / or 1x5mL lithium Heparin blood tube for LPV/r arms).

8 hours post first dose:

- *For sites involved in the PK component:* Research blood sample (1x5mL EDTA for hydroxychloroquine arms and / or 1x5mL lithium Heparin blood tube for LPV/r arms).

2.5.5.3 Day 2

These activities will be performed whilst the participant is in hospital and prior to study drug dosing:

- Vital signs;
- Review of clinical observations, including any adverse events;
- ICU status;
- Prior to the 3rd dose of study drug the 24hour ECG post initial dose should be performed;
- A blood glucose level check via a finger prick to be performed prior to the 24hour ECG;
- Review of routine clinical blood test results including full blood examination (FBE), biochemistry (i.e. EUC, LFTs, CRP);
- Clinical blood test, not mandated but recommended, K+ and Mg2+. *If K+ or Mg2+ are abnormal, then they should be corrected*
- Study drug administration doses and times recorded.

If the participant is discharged either today or any time during the study after this time point, then the site PI or their delegate will contact the participant by phone on days 8, 11, 16 and 28 as outlined in the Schedule of events (table 4) with the exception of weekends and public holidays. The purpose of this contact is to check compliance with the protocol in terms of study drug prescribing and recording any adverse events.

After the 4th dose of study drug and only for sites involved in the PK component the following samples will be taken:

- Research blood sample (1x5mL EDTA for hydroxychloroquine arms and / or 1x5mL lithium heparin blood tube for LPV/r arms) ***4 hours*** post 4th (or 5th) study dose;
- Research blood sample (1x5mL EDTA for hydroxychloroquine arms and / or 1x5mL lithium heparin blood tube for LPV/r arms) ***8 hours*** post 4th (or 5th) dose;

The PK samples may be taken on day 3 after the 5th dose of study drug, if more practicable for the site staff.

2.5.5.4 Day 3

These activities will be performed if the participant is in hospital:

- Review of vital signs;
- Review of routine clinical blood test results including full blood examination (FBE), biochemistry (i.e. EUC, LFTs, CRP);
- Clinical blood test, not mandated but highly recommended, lactate, D-dimer, LDH, ferritin, coagulation studies (APTT, INR, fibrinogen), troponin, BNP;
- Review of clinical observations, including any adverse events;
- Review of ICU status;
- Review of routine clinical respiratory tract swab results (if applicable);
- Chest imaging. A CXR or CT chest is not mandated investigation, and data will only be collected if this has been performed as part of routine care on day 3 or results from the preceding 24-hour period;
- Study drug administration doses and times recorded.
- *For sites involved in the enhanced biological component (tier 1):*
    - Research blood sample (1x9mL SST blood tube);
- *For sites involved in the research biological component (tier 2):*
    - Research blood sample (3x9mL sodium heparin tubes and 2x9mL ACD tubes);

2.5.5.5 Days 4, 5, 6, 8, 9

These activities will be performed if the participant is in hospital:

- 12 lead ECG;
- Review of vital signs;
- Review of clinical observations, including any adverse events;
- ICU status;
- Study drug administration doses and times recorded;

If the participant was discharged before Day 7, then the PI or their delegate will contact the participant by phone on ***Day 8*** and check:

- Current health status;
- Number of study drug tablets left;
- Targeted side effects (i.e. diarrhoea, nausea, vomiting);
- Any shortness of breath experienced.

2.5.5.6 Day 7

These activities will be performed if the participant is in hospital:

- Review of vital signs;

- Review of clinical observations, including any adverse events;
- Review of routine clinical blood test results including full blood examination (FBE), biochemistry (i.e. EUC, LFTs, CRP);
- Clinical blood test, not mandated but highly recommended, lactate, D-dimer, LDH, ferritin, coagulation studies (APTT, INR, fibrinogen), troponin, BNP;
- ICU status;
- Review of routine clinical respiratory tract swab results;
- Chest imaging. A CXR or CT chest is not mandated investigation, and data will only be collected if this has been performed as part of routine care on day 7 or results from the preceding 24-hour period;
- Study drug administration doses and times recorded;
- *Only for sites involved in the enhanced biological component (tier 1):*
    - Research blood sample (1x9mL SST blood tube);
    - Respiratory tract sample (Nasopharyngeal and throat swab) for biobanking;
- *For sites involved in the research biological component (tier 2):*
    - Research blood samples (3x9mL sodium heparin tubes and 2x9mL ACD tubes).

2.5.5.7 Day 10

These activities will be performed if the participant is in hospital:

- Review of vital signs;
- Review of clinical observations, including any adverse events;
- ICU status;
- Review of routine clinical blood test results including full blood examination (FBE), biochemistry (i.e. EUC, LFTs, CRP);
- Clinical blood test, not mandated but highly recommended, lactate, D-dimer, LDH, ferritin, coagulation studies (APTT, INR, fibrinogen), troponin, BNP;
- Study drug administration doses and times recorded;

2.5.5.8 Days 11-14

These activities will be performed if the participant is still in hospital:

- Review of clinical observations, including any adverse events;
- ICU status.

If the participant was discharged before Day 10 then the PI or their delegate will contact the participant by phone on ***Day 11*** and check:

- Current health status;
- Number of study drug tablets left;
- Targeted side effects (i.e. diarrhoea, nausea, vomiting);
- Any shortness of breath experienced.

2.5.5.9 Day 15

These activities will be performed if the participant is in hospital:

- Review of clinical observations, including any adverse events;
- Review of routine clinical blood test results including full blood examination (FBE), biochemistry (i.e. EUC, LFTs, CRP);

- Clinical blood test, not mandated but highly recommended, lactate, D-dimer, LDH, ferritin, coagulation studies (APTT, INR, fibrinogen), troponin, BNP;
- *Only for sites involved in the enhanced biological component (tier 1):*
  - Research blood sample (1x9mL SST blood tube);
- *For sites involved in the research biological component (tier 2):*
  - Research blood samples (3x9mL sodium heparin tubes and 2x9mL ACD tubes.

If the participant was discharged before Day 15, then the PI or their delegate will contact the participant by phone on ***Day 16*** and check:

- Current health status;
- Targeted side effects (i.e. diarrhoea, nausea, vomiting);
- Any shortness of breath experienced.

2.5.5.10 Day 28 + 3 days

These activities will be performed if the participant is in hospital:

- Clinical observations, including any adverse events;
- *Only for sites involved in the enhanced biological component (tier 1):*
  - Research blood sample (1x9mL SST blood tube);
- *For sites involved in the research biological component (tier 2):*
  - Research blood samples (3x9mL sodium heparin tubes and 2x9mL ACD tubes).

If the participant has been discharged prior to Day 28, then the site PI or their delegate will contact the participant by phone on ***Day 28*** and record the following information:

- participant status,
- current oxygen use (if applicable)

2.5.5.11 Day 90 + 14 days

At Day 90 ***or later***, site staff will review hospital medical records and/or contact the participant (or GP) to record the following information:

- Participant status – alive or dead;
- Date of death (if applicable);
- Current oxygen therapy (if applicable)

2.5.6 Data and sample collection

CRFs will be filled out within 48 hours of the relevant day. For example, the day 1 CRF will be filled out on day 2–3, day 3 on day 4–5 and day 7 on days 8–9.

There will be four tiers for collection of research samples and associated data. All sites will need to collect core clinical data. Sites may opt-in to different parts of the enhanced biological, research biological or PK testing tiers.

2.5.6.1 Core Data

These are the core clinical data that contribute to the primary endpoint, key secondary outcomes, and key potential confounders. The procedures/investigations undertaken and recorded as part of this clinical data set are part of routine care – except for the ECGs taken at 4hrs, 24hrs and day 4 post initial drug administration for the first 100 participants.

2.5.6.2 Enhanced biological (tier1)

Includes research blood, stool and respiratory sampling.

Blood and stool samples for the analysis of potential biomarker assays, immunological responses including but not limited to antibody levels, response to virus and genetic analysis including genome wide association study (GWAS).

Genomics is increasingly informing researcher's understanding of disease pathobiology. Large-scale human GWAS studies require large collections of DNA from people exposed to, or infected with, SARS-CoV-2. To identify human genes related to susceptibility or disease outcome, this would require COVID-19 individuals with well-defined clinical phenotypes, as GWAS are case-control studies that compare phenotypes, i.e. mild disease vs severe disease, asymptomatic vs active disease, survivors vs non-survivors. To ensure large sample sizes of individuals with differing clinical phenotypes it is essential to collect DNA from multiple studies, with diverse study designs. For example, DNA from active COVID-19 cases from the ASCOT study and from asymptomatic exposed individuals from household or population studies.

2.5.6.3 Research biological (tier 2)

Includes additional blood sampling for PBMC isolation and biobanking. Sites that agree to participate in tier 2 sampling, should also have tier 1 samples collected.

2.5.6.4 PK sampling

Blood samples for measurements of serum concentrations of lopinavir/ritonavir and/or hydroxychloroquine will be collected at timepoints specified in Table 4. Sites can opt to be involved in PK sampling, without being involved in tiers 1 or 2 research sampling or be involved in addition to tiers 1 & 2 research sampling.

2.5.7 Discontinuation/Withdrawal of participants from trial treatment

The participants have the right to choose to withdraw from the study at any time and the investigator may discontinue a participant from the study or from treatment if deemed appropriate at any time. Reasons why a participant may be withdrawn from the study include, but are not limited to:

- participant request,
- primary treating clinicians request,
- participant was enrolled and is ineligible (either arising during the study or was overlooked at time of screening and enrolment).

Participants will not be withdrawn due to adverse events. The decision to withdraw a participant from the study must be discussed with the coordinating investigators.

If the participant withdraws consent from participating in the study and also withdraws consent for disclosure of future information, no further evaluations will be performed, and no additional data will be collected. The co-ordinating investigators may retain and continue to use any data or samples collected before such withdrawal of consent. Participants that leave against medical advice will continue to be followed until the end of the trial to avoid missing data, if they did not complete treatment their data will be used in the intention-to-treat analysis, if they completed treatment their data will be used in the as per protocol analysis. Participants that are lost to follow up will continue to be followed until the end of the trial to avoid missing data, if the participant completed treatment their data will be used in the as per protocol analysis. Participants withdrawn from the treatment by the treating

clinicians will continue to be followed up to the end of the trial to avoid missing data and will be used in the intention-to-treat analysis.

If a participant is withdrawn the reason will be recorded in the database.

The study drug the participant is randomised to will be discontinued if a participant chooses to withdraw from the study.

If a participant is admitted to ICU due to progressive disease, the treating clinician can continue treatment using the allocated study drug for a total of 10 days. At the time of ICU admission, ASCOT participants meet the study primary endpoint, and may thereafter be enrolled in other clinical trials if eligible, including randomisation to other interventions or continuation of ASCOT study drugs according to study investigators.

2.5.8 End of trial

At this stage this is an open-ended study given the uncertainties of the epidemiological course of SARS-CoV-2. The trial steering committee will continually assess the epidemiological situation, trial progress and interim results, and emerging external evidence of efficacy of the study and other interventional agents. The trial protocol may be adapted as the situation changes.

2.6 Study timeline

This project will aim to commence as soon as possible in 2020.

Figure 2 – Study timelines

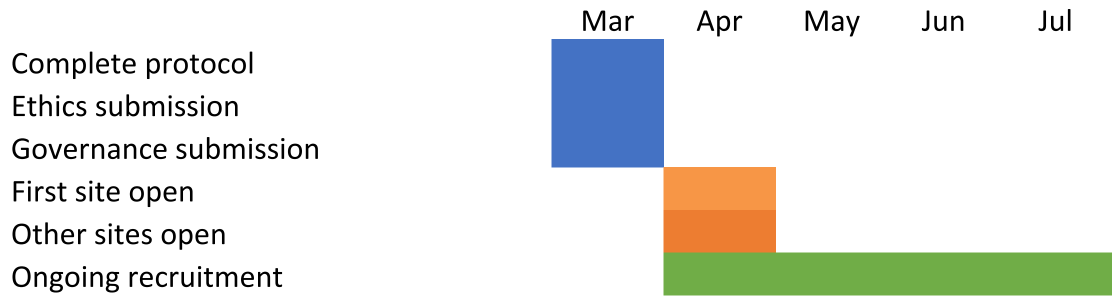

2.7 Sample size

There is great uncertainty at this early stage of the epidemic. Until further information becomes available there is not sufficient data to predict likely numbers of patients presenting with COVID-19.

2.7.1 Primary endpoint

Estimated rates of need for ventilation among hospitalised patients have varied. In an early report from China of 1099 hospitalised cases, 5% required ICU admission with 2.3% undergoing invasive ventilation [1]. In Italy, ICU admissions (n=556) represented 16% of all patients (n=3420) who tested positive for COVID-19 [11].

The following table provides an example of a 2x2 factorial design assuming relative risk 0.5 by LPV/r and by hydroxychloroquine (HCQ) and no interaction between LPV/r and HCQ. Percentages in each cell are the event rate.

	HCQ - Yes	HCQ – No	Total – main comparison for HCQ
LPV/r – Yes	1.25%	2.5%	1.875%
LPV/r – No	2.5%	5% (standard)	3.75%
Total – main comparison for LPV/r	1.875%	3.75%	

Given the 2x2 factorial design, each main comparison (either LPV/r or HCQ) is comparing 3.75% vs 1.875% (across rows, or across columns), then the sample size would be 610 per arm (per cell) with a two-sided significance level (alpha) of 5% and 80% power. The total sample size would be 2440 allowing for two comparisons.

We will use the Haybittle-Peto approach for interim analyses. Other rules (e.g., O'Brien-Fleming), while preferable in standard settings, require specification of the number and timing of interim analyses which does not allow for sufficient flexibility at this stage. We note that the Haybittle-Peto approach will be an overly conservative approach throughout, necessary in this situation.

The calculations in the below table are for power of 70%, 80% and 90%, and for alpha of 0.05 and 0.045. The 0.45 value is investigated as a conservative estimate to adjust for the interim analyses according to the Haybittle-Peto rule, without knowing a priori the number, timing and type of the interim analyses (i.e., efficacy/ harm/ futility). Numbers refer to total and in each study arm.

	70%	80%	90%
0.045	1988 (497)	2520 (630)	3356 (839)
0.05	1920 (480)	2440 (610)	3264 (816)

The below table provides estimated sample sizes for different proportions of hospitalised patients needing ventilation.

For a reduction in the primary endpoint from 10% to 5%:

	70%	80%	90%
0.045	968 (242)	1224 (306)	1628 (407)

For a reduction in the primary endpoint from 15% to 10%:

	70%	80%	90%
0.045	1376 (344)	1740 (435)	2320 (580)

2.8 Assignment of interventions

2.8.1 Allocation

Participants will be randomised in a 1:1:1:1 ratio to active treatment or standard of care treatment arms, using a randomisation schedule generated by an independent statistician

produced and uploaded into the database which is available 24 hours per day, 7 days per week.

Randomisation will be stratified by site and will be in permuted blocks of variable block size.

2.8.2 Allocation concealment

The randomised sequence allocation will be stored in the database and will not be available to any investigators or member of study staff.

2.8.3 Implementation

The allocation sequence will be generated by a statistician not involved in the day to day trial procedures. Participants will be enrolled by site principal investigators or their delegates (research nurse or co-investigator). The person enrolling the participant will, following obtaining verbal informed consent, obtain the treatment allocation by logging onto the electronic data capture system, completing the required fields before the system will allow randomisation and subsequent treatment allocation.

2.8.4 Blinding

This is an open-label study, but researchers assessing the laboratory outcomes will be blinded to treatment allocation. Although blinding was considered, the added complications and expense were deemed prohibitive. Furthermore, no placebo of LPV/r was available from AbbVie at the time of trial design.

2.9 Data Management and Quality Assurance

2.9.1 Source data

Source documents are where data are first recorded, and from which participants' CRF data are obtained. These include but are not limited to, hospital records both electronic & paper (which will include medical history, previous and current medications, any relevant radiography test, blood test results, haemodynamic parameters and medical correspondence) and electronic clinic records (which will include vital status, recent medical history and relevant blood culture results). A further data source will be through telephone conversations with the study participant or GP.

Storage and archiving of study documents (CRFs and consent forms) will be the responsibility of the site principal investigator and will remain at the site of recruitment and retained for 15 years. All study participants will be allocated a unique number at time of screening (screening number), this screening number will be added to all the CRFs for that participant. The participants will also have their HRN recorded on the CRFs as this information will be required to ensure the correct medical record is accessed during medical record reviews. The date and time will be captured on the CRF for all telephone conversations with study participants or GP.

2.9.2 Protocol Deviations and Serious Breaches

Protocol deviations will be recorded in the participant record (source document) and on the CRF and must be reported to the Site Principal Investigator, who will assess for seriousness. In this study, protocol deviations collected for this study will be limited to:

- Missed visits as outlined in the Schedule of Events Table 4;
- >20% of study drug doses missed;
- Incorrect dosing of study drug;
- Day 15 outcomes missing.

Those deviations deemed to affect to a significant degree rights of a trial participant or the reliability and robustness of the data generated in the clinical trial will be reported as serious breaches. Reporting will be done in a timely manner (Site Principal Investigator to report to the Sponsor within 72 hours and to the Site RGO within 7 days; Sponsor to review and submit to the approving HREC within 7 days).

Where non-compliance significantly affects human participant protection or reliability of results, a root cause analysis will be undertaken and a corrective and preventative action plan prepared.

Where protocol deviations or serious breaches identify protocol-related issues, the protocol will be reviewed and, where indicated, amended.

2.9.3 Data Recording and Record Keeping

Data for this study will be recorded via a secure, Electronic Data Capture (EDC) web-based system using the eCRFs. It will be transcribed by the site PI or their delegate from the paper CRFs onto the eCRF (in no case is the eCRF to be considered as source data for this trial). Data will be stored in a re-identifiable manner in the database, using a unique screening number for each patient.

The database will contain validation ranges for each variable to minimise the chance of data entry errors. An audit trail will maintain a record of initial entries and changes made; reasons for change; time and date of entry; and username of person who made the change. Data queries will be raised by the project manager and study monitor, and missing data or suspected errors will be raised as data queries and resolved prior to database lock and analysis. The database will contain in-line capability so that these queries and answers are logged as part of the audit trail.

For each potential participant screened (even those who are not eligible), the screening eCRF will be completed by the site PI or their delegate. For each participant enrolled, eCRFs must be completed. This also applies to records for those patients who fail to complete the study. The site PI should ensure the accuracy, completeness and timeliness of the data reported to the sponsor in the eCRFs and in all required reports. A comprehensive validation check program will verify the data and automatically generate discrepancies for resolution by the investigator. Manual discrepancies can also be raised if necessary.

In addition, accurate and reliable data collection will be assured by verification of the eCRFs against the investigator's records by the study monitor (source document verification), and the maintenance of medication compliance will be captured in the CRFs from the participant's medication chart (source document) by the investigator.

Storage and archiving of hard-copy study documents (CRFs and consent forms) will be the responsibility of the principal investigator at each site and will remain at the site of recruitment following local security guidelines. Hard-copy study documents will be kept for a

minimum of 15 years and confidentially destroyed at the end of this period only with the express consent of the study sponsor.

2.9.4 Confidentiality

Participant confidentiality is strictly held in trust by the participating investigators, research staff, and the sponsoring institution and their agents. This confidentiality is extended to cover testing of biological samples and genetic tests in addition to the clinical information relating to participating participants.

The trial protocol, documentation, data and all other information generated will be held in strict confidence. No information concerning the trial or the data will be released to any unauthorised third party, without prior written approval of the sponsoring institution. Authorised representatives of the sponsoring institution may inspect all documents and records required to be maintained by the Investigator, including but not limited to, medical records (office, clinic or hospital) and pharmacy records for the participants in this trial. The clinical trial site will permit access to such records.

All laboratory specimens, evaluation forms, reports and other records that leave the site will be identified only by the Participant Identification Number to maintain participant confidentiality.

Clinical information will not be released without written permission of the participant, except as necessary for monitoring by HREC or regulatory agencies.

2.10 Statistical methods

2.10.1 Statistical analysis plan

Data will be reported in accordance with the CONSORT guidelines for reporting of randomised trials. Proportions will be compared between treatment groups with Fisher's exact or χ^2 tests, and the absolute difference in proportions reported with corresponding 95% confidence intervals. All-cause mortality will be presented in a Kaplan-Meier graph.

The ***primary analysis*** of both primary and secondary endpoints will be according to modified intention to treat principles (all participants with data available for the endpoint will be analysed according to the treatment allocation, regardless of what treatment they received). No assumptions will be made about those with missing data.

A ***secondary per-protocol analysis*** of all endpoints will be conducted. The per protocol population is defined as 1) for the control group: did not receive any LPV/r or hydroxychloroquine; 2) for the intervention group: received at least 80% of possible doses of study drug; 3) has available data.
We will perform the following **subgroup analyses**:

1. ***Age ≥65 or <65 years***. Early experience with SARS-CoV-2 infection is that older age is associated with poorer outcomes.
2. ***Receipt of study drug within or after 96 hours of symptom onset***. There is biological plausibility that early treatment to reduce viral replication will be more effective than later treatment when pulmonary pathology may already be evident.

3. ***Mild vs moderate severity at presentation***. Initial severity at presentation may predict the later clinical course and there may be a differential effect of antiviral treatment. The absolute improvement in clinical outcomes may be more evident in the moderate severity group due to a higher event rate.
   a. Mild severity at presentation is defined as: SaO2≥95% on room air AND not requiring supplemental oxygen AND not tachypnoeic (respiratory rate <24 breaths/min)
   b. Moderate severity at presentation is defined as SaO2≤94% on room air OR requiring supplemental oxygen OR tachypnoeic (respiratory rate ≥24 breaths/min)
4. ***Baseline use of ACE inhibitors or ATIII blockers***. These medications affect the renin angiotensin aldosterone system pathway, which may also be affected by SARS-CoV-2, which uses ACE2 as its cellular receptor. Use of these medications may result in differential susceptibility to SARS-CoV-2 infection, with the direction of effect uncertain.
5. ***Those with baseline immunosuppression vs those without*.** These are different patient groups with regards to underlying comorbidities and risk for severe sepsis. Patients will be considered immunosuppressed if in receipt of immunosuppressing medication considered by the site investigator to be equivalent to ≥20 mg of prednisolone for ≥2 weeks, or with a known haematological malignancy.
6. ***Those with end stage kidney disease (ESKD, meaning receipt of haemodialysis or peritoneal dialysis) at enrolment.*** The pharmacokinetics of hydroxychloroquine are uncertain in this population, the risk of drug-related adverse events may be higher, and the efficacy of the study drugs maybe either better (higher blood levels) or worse (underlying comorbidities leading to poor outcomes regardless of adjunctive therapies).
7. ***Those who experienced acute kidney injury any time between randomisation and day 15.*** The pharmacokinetics of hydroxychloroquine are uncertain in this population, the risk of drug-related adverse events may be higher, and the efficacy of the study drugs maybe either better (higher blood levels) or worse (underlying comorbidities leading to poor outcomes regardless of adjunctive therapies).

2.10.2 Interim analyses and stopping guidelines

The Data and Safety Monitoring Board (DSMB) will not conduct an interim analysis until a decision has been made on whether to convert to an adaptive platform trial framework. If it is decided to not covert to an adaptive platform trial framework, an interim analysis can occur after 200 participants have been randomised. There will be continued review of the need for further interim analyses.

The interim analysis will review outcome data and answer the following questions:

1. Are there any significant safety issues that may present an ethical issue in continuing the study? This may include adverse events, but also study conduct and protocol violations
2. Is there overwhelming data suggesting the superiority of one arm that may present an ethical issue in continuing the study? The interim analyses will be adjusted according to Haybittle-Peto, separately for the LPV/r and HCQ main effects, using an overall two-sided 5% significance level across the interim and final analyses for each main effect.

Should the result for a particular main effect (e.g., LPV/r vs no LPV/r) cross the designated boundary at an interim analysis, consideration will be given to termination of the study of that intervention (e.g., cease recruitment to the two LPV/r arms and randomize new patients to HCQ or Standard care only). Details will be provided in the DSMB charter.

3. Are there any other factors that may impact on the feasibility / usefulness of the study? E.g., rate of enrolment, unexpected low rate of outcomes, unable to fund, protocol violations etc.
4. Should the study continue in light of emerging data on treatment of SARS-CoV-2?

In addition to the planned interim analyses, the DSMB will monitor the emerging literature on the effect of antiviral and other treatments on COVID-19. If data are published which demonstrate that one of the trial interventions (LPV/r or hydroxychloroquine) are clearly superior to standard of care, then the DSMB will consider recommending dropping the standard of care arm or ceasing the trial entirely. A study with three treatment arms would address the comparative benefit of LPV/r vs hydroxychloroquine vs both drugs combined. If either LPV/r or hydroxychloroquine are clearly harmful, then the relevant treatment arms could be dropped. The trial steering committee will make the final decision on these matters.

After 100 participants have been randomised, the DSMB will review data on QTc and gastrointestinal tolerability of study drug regimens. Recommendations can then be made as to safety of the study drug regimens and what ongoing monitoring requirements will be for subsequent patients. In particular, a recommendation will be sought for whether an ECG 4 hours post initial drug dose is still required. The primary endpoint data will not be assessed at this review after 100 participants.

2.10.3 Provision for a Bayesian adaptive trial approach

The trial is currently designed with a frequentist analysis framework. This approach has been necessary given the time constraints in designing and commencing recruitment as soon as possible. However, the trial steering committee will consider moving to a Bayesian adaptive trial approach after further consultation. No interim analyses will be performed until such a decision has been made. If it is decided to convert to a Bayesian adaptive trial approach, no interim analyses will be performed until a pre-specified Bayesian adaptive analysis plan has been designed and agreed upon.

2.11 Monitoring and trial co-ordination

2.11.1 Trial co-ordination

This trial will be centrally co-ordinated from the Doherty Institute. The study will also have input from the Australasian Society for Infectious Diseases Clinical Research Network.

2.11.2 Data safety and monitoring board (DSMB)

An independent DSMB will be established to review the progress of the study and monitor adherence to the protocol, participant recruitment, outcomes, complications, and other issues related to participant safety. They will also monitor the assumptions underlying sample size calculations for the study and alert the investigators if they see substantial departures as

the data accumulate. A copy of recommendations from the DSMB will be sent to respective HRECs.

The DSMB will be composed of experts in infectious diseases, biostatistics, clinical trials, virology and immunology. The DSMB members will all be independent of the investigators (none of them will be chief investigators or site investigators).

The DSMB will make recommendations as to whether the study should continue or be terminated, consider participant safety or other circumstances as grounds for early termination, including either compelling internal or external evidence of treatment differences or feasibility of addressing the study hypotheses (e.g. poor participant enrolment, poor adherence).

The DSMB will operate under the rules of an approved charter that will be written and reviewed at the organisational meeting of the DSMB.

2.11.3 Study monitoring

Study monitoring will be provided by the responsible monitor(s) in accordance with the Monitoring Plan and principles of ICH GCP. The monitoring plan will be developed and will likely mainly rely on central monitoring with limited site visits depending on resources and number of sites.

2.12 Safety

All study drugs are licensed for use in Australia with established safety profiles.

An Adverse Event (AE) is defined as any untoward medical occurrence in a patient or clinical trial participant administered a medicinal product/s that does not necessarily have a causal relationship with this treatment. An AE can therefore be any unfavourable and unintended sign (including an abnormal laboratory finding), symptom, or disease temporally associated with the use of the study drug, whether or not causally linked to the investigational drug.

2.12.1 Adverse Reaction (AR) definition

An adverse event that is judged by the reporting medically qualified professional as having a reasonable suspected causal relationship with the trial drug.

2.12.2 Reference Safety Information (RSI) definition

The information contained in an approved Australian Product Information contains the information used to determine what adverse reactions are to be considered expected adverse reactions and, on the frequency, and nature of those adverse reactions.

2.12.3 Safety Critical Adverse Events

For this study, specific adverse events (refer to section 2.4.2) will be collected for all participants as pre-defined secondary outcomes and are defined in Table 5. Enzyme investigations and imaging only required for pancreatitis if clinically indicated.

Table 5: Definition of adverse events

	Grade 1	Grade 2	Grade 3	Grade 4
Diarrhoea	Increase of <4 stools per day over baseline; mild increase in ostomy	Increase of 4 - 6 stools per day over baseline; moderate increase in ostomy output compared to	Increase of >=7 stools per day over baseline; hospitalization indicated; severe	Life-threatening consequences; urgent intervention indicated

	Grade 1	Grade 2	Grade 3	Grade 4
	output compared to baseline	baseline; limiting instrumental ADL	increase in ostomy output compared to baseline; limiting self-care	
Nausea	Loss of appetite without alteration in eating habits	Oral intake decreased without significant weight loss, dehydration or malnutrition	Inadequate oral caloric or fluid intake; tube feeding, TPN, or hospitalization indicated	-
Vomiting	Intervention not indicated	Medical intervention indicated	Tube feeding, TPN, or hospitalization indicated	Life-threatening consequences
Pancreatitis	-	Enzyme elevation; radiologic findings only	Severe pain; vomiting; medical intervention indicated (e.g., analgesia, nutritional support)	Life-threatening consequences; urgent intervention indicated

2.12.4 Serious adverse events (SAEs)

For this study, a SAE is defined as any adverse event that:

- Results in death
- Is life-threatening
  The term "life threatening" refers to an event in which the participant was at risk of death at the time of the event. It does not refer to an event, which hypothetically may have caused death, if it were more serious.
- Results in unexpected prolongation of existing hospitalisation
- Results in persistent or significant disability/incapacity
- Is a medically important event or reaction

Note: *Medical and scientific judgement should be exercised in deciding whether an adverse event/reaction should be classified as serious in other situations. Important medical events that are not immediately life-threatening or do not result in death or hospitalisation but may jeopardise the participant or may require intervention to prevent one of the other outcomes listed in the definition above should also be considered serious*.

For this study we will be using the Common Terminology Criteria for Adverse Events (CTCAE) version 5
https://ctep.cancer.gov/protocoldevelopment/electronic_applications/docs/CTCAE_v5_Quick_Reference_5x7.pdf

SAEs are monitored continuously and have special reporting. Sites are not required to report SAEs which are considered to be consistent with COVID-19 and expected disease progression including:

- Hypoxia;
- increased requirement for supplemental oxygen;
- mechanical ventilation;
- ICU admission or death due to respiratory failure;
- venous thromboembolism;
- chest pain;
- shortness of breath;

- fever or other features judged clinically to be consistent with COVID-19 disease progression.

This study is only collecting SAEs for any of the adverse events listed below, if thought to be attributable to one or more of the study drugs from randomisation up until 30 days post last dose of study drug:

SAEs of special interest
For LPV/r:

- Acute pancreatitis
- Hepatotoxicity with evidence of failure
- Anaphylaxis or other suspected serious immune-mediated reaction
- Life-threatening arrythmia requiring administration of an anti-arrhythmic medication, cardioversion, or any form of cardiac pacing.

For hydroxychloroquine:

- Severe hypoglycaemia
- Anaphylaxis or other suspected serious immune-mediated reaction
- Life-threatening arrhythmia requiring administration of an anti-arrhythmic medication, cardioversion, or any form of cardiac pacing

Where clinically appropriate, study drug should be ceased.

2.12.5 Suspected Unexpected Serious Adverse Reaction (SUSAR) definition

Consider a SUSAR as any SAE that is both suspected to be related to the study drug treatment and is unexpected (i.e. not consistent with the available RSI contained in the approved Product Information).

2.12.6 Significant Safety Issue (SSI)

A safety issue that could adversely affect the safety of participants or materially impact on the continued ethical acceptability or conduct of the trial.

A SSI is a new safety issue or validated signal considered by the Sponsor in relation to the study drug(s) that requires urgent attention. This may be because of the seriousness and potential impact on the benefit-risk balance of the investigational study drug, which could prompt regulatory action and/or changes to the overall conduct of the clinical trial, including the monitoring of safety and/or the administration of the study drug product

2.12.7 Urgent Safety Measurement (USI)

A measure required to be taken in order to eliminate an immediate hazard to a participant's health or safety.

This is a type of SSI that can be instigated by either the investigator or sponsor and can be implemented before seeking approval from HRECs or institutions

2.12.8 Causality

The principal site investigator will make a judgement regarding whether an adverse event is clinically significant and whether or not it is related to the allocated treatment. The degree of certainty with which an adverse event is attributable to treatment or an alternative cause will be determined by how well the event can be understood in terms of:

- Temporal relationship with the administration of the treatment or cessation of treatment
- Reactions of a similar nature previously observed in the individual or others following treatment

The relationship of the adverse event to treatment will be specified as follows:

Not related	In the PI's opinion, there is not a causal relationship
Unlikely	The temporal association between treatment and the adverse event is such that treatment is not likely to have any reasonable association.
Possibly	The adverse event could have been caused by treatment.
Probably	The adverse event follows a temporal sequence from the time of treatment and cannot be reasonably explained by the known characteristics of the participant's clinical presentation/history.
Definitely	The adverse event follows a reasonable temporal sequence from the time of treatment or reappears when the treatment is repeated.

2.12.9 Summary reporting of adverse events and adverse drug reactions

Site Principal Investigator Reporting Procedures:

The Site PI is responsible for recording all safety events in the source document.

The Site PI is responsible for expedited reporting (within 24 hours of becoming aware of the event) to the Sponsor the following local safety events:

- USMs
- SUSARs
- All SAEs except:
  - Those not thought to be related to the study drug (e.g. death from respiratory failure – expected for COVID-19);

The Site PI is also responsible for reporting SSIs, local USMs and local SUSARs to their research governance office within 72 hours of becoming aware of the event and in accordance with their local governance authorization.

The minimum amount of information that the site investigator must complete on the initial report is:

- title of the event – avoid colloquialisms or abbreviations;
- date started;
- reason the event is considered an SAE;
- causality relationship to investigational drug/device.

The PI or designee should complete the SAE form and send to:

BaCT Safety team: Safety_BaCT@petermac.org
ASCOT Coordinating Centre: ascot-team@unimelb.edu.au

Sponsor Reporting Procedures:

The Sponsor must assess and categorise the Safety Reports received from Investigators and report these to all Site Principal Investigators, the approving HREC and TGA in accordance with the NHMRC's 'Safety monitoring and reporting in clinical trials involving therapeutic goods' (November 2016) and any additional requirements of the approving HREC. All safety

reports must clarify the impact of the safety event on participant safety, trial conduct and trial documentation.

The Sponsor is responsible for the following reporting to PIs, the HREC(s) and TGA:

1. All SSIs that meet the definition of a USM within 72 hours of becoming aware of the issue.
2. All other SSIs within 15 calendar days of instigating or becoming aware of the issue
3. For SSIs leading to an amendment of trial documentation:
    a. Submit details of the SSI without undue delay and no later than 15 calendar days of becoming aware of the issue.
    b. Submit amendment to the HREC without undue delay.
4. For SSIs leading to temporary halt or early termination of a trial for safety reasons:
    a. Communicate reasons, scope of halt, measures taken, further actions planned without undue delay and no later than 15 calendar days of decision to halt.
    b. For a temporary halt, notify the PIs, HREC and TGA when the trial restarts, including evidence that it is safe to do so.

The Sponsor will also report SUSARs to the TGA as follows:

1. Fatal or life-threatening SUSARs immediately, but no later than 7 calendar days after being made aware of the issue (follow up info within a further 8 calendar days)
2. All other SUSARs no later than 15 calendar days of being made aware of the issue

The Sponsor is responsible for providing information to the approving HREC and to investigators of any updated Product Information for the study drugs.

2.13 Ethical considerations

2.13.1 General ethical considerations

The study will be conducted according to the declaration of Helsinki, the NHMRC criteria for the ethical conduct of research in humans and the principles of Good Clinical Practice [12].

The therapeutic agents lopinavir/ritonavir (LPV/r) oral solution and hydroxychloroquine used in this study are registered for use in Australia and New Zealand. The Mylan brand of LPV/r has only been registered in the EU. It is currently in the process of being registered in the US through the FDA.

LPV/r has proven safe in the HIV setting. Hydroxychloroquine is widely used for autoimmune conditions. Chloroquine is widely used as an anti-malarial agent. Approval will be sought from relevant human research ethics committees (HRECs) for all sites.

The study protocol, information statements, consent forms, and any other documents required for ethics approval will be submitted to the relevant HRECs for approval before the study commences. Each HREC reviewing the protocol must be properly constituted according to NHMRC requirements and have the capacity to review the study. Approvals must specify the study title, version numbers, and identify all documents reviewed and state the date of review. No amendments to, or deviations from, the protocol must be initiated without prior written approval from the relevant HREC. The exceptions to this are:

- administrative aspects that have no bearing on participants;
- the need to address regulatory requirements; and/or,

- the need to eliminate immediate hazards to the participants.

The Sponsor will inform the HREC of the following:

- all protocol amendments, informed consent changes or revisions of other documents originally submitted for review;
- serious and/or unexpected adverse events
- new information that may affect the safety of the participants or the proper conduct of the trial;
- annual updates of study progress
- termination of the study including provision of a final study report.

2.13.2 Summary of potential harms and risks to study participants

Both LPV/r and Hydroxychloroquine have been used in millions of people worldwide over decades, and have established safety profiles. However, with both of these drugs there is a small risk of severe and possibly even fatal adverse events (see section 1.2.4 for more detail). We believe this small risk is justified because of the potential for benefit, and the context that the enrolled patients have a serious infection with has a 1.5%-7% chance of mortality (hundreds of times higher than the risk of severe AEs from the study drugs). Furthermore, eligibility criteria and monitoring mitigate the risk of severe AEs, and patients have provided informed consent, with the knowledge of the possibility of these AEs.

2.13.3 Informed consent

See Section 2.5.3.

2.13.4 Drug shortages

In the event of shortages of either of the investigational product LPV/r and hydroxychloroquine at participating trial sites, then treatment arms involving that drug will be suspended at those sites. Randomisation to standard of care or monotherapy with the remaining drug will continue. If both drugs are not available at participating trial sites, then the trial will be suspended at those sites.

The trial steering committee will liaise closely with drug providers to secure drug supply during the course of the study.

2.14 Regulatory approvals

Although LPV/r and hydroxychloroquine are licensed for use in Australia and New Zealand, they will be used outside their approved indications. Hence a Clinical Trials Notification (CTN) will be lodged with the Therapeutic Goods Administration (TGA) for all Australian sites.

2.15 Data harmonisation, access and sharing

2.15.1 Data harmonisation

Given the importance of data sharing with other studies being concurrently conducted, data collection and protocols are being harmonised with:

- WHO Master Protocol: https://www.who.int/blueprint/priority-diseases/key-action/multicenter-adaptive-RCT-of-investigational-therapeutics-for-COVID-19.pdf

- ISARIC (International Severe Acute Respiratory and Emerging Infection Consortium) WHO case Record Form: https://isaric.tghn.org/covid-19-clinical-research-resources/
- REMAP-CAP (A Randomised, Embedded, Multi-factorial, Adaptive Platform Trial for Community-Acquired Pneumonia): https://www.remapcap.org/

2.15.2 Data Access

The trial steering committee will be the custodians of the final trial dataset. No-one outside the trial steering committee will be given access to the data without the permission of the trial steering committee. No identifying data will be given to any third parties at any stage. Following study close out and locking of the database, it will be stored on the servers of the sponsor.

2.15.3 Data sharing

2.15.3.1 *Global datasets*

Harmonisation of data collection as detailed in 2.15.1 should facilitate data sharing. The study will have an ethos of appropriate sharing of data to contribute towards global datasets.

2.15.3.2 *Communication with REMAP-CAP*

At study sites where REMAP-CAP is open for recruitment, participants enrolled in ASCOT and progressing to ICU admission or need for invasive and non-invasive ventilation will be identified to REMAP-CAP study staff as being enrolled in ASCOT. The ASCOT unique study number will be communicated to REMAP-CAP to allow linkage of data.

2.15.3.3 *Communication with SPRINT-SARI*

At study sites where SPRINT-SARI is collecting observational data, data from participants enrolled in ASCOT can be shared with SPRINT-SARI. No identifiable data will be provided.

2.15.3.4 *Communication with participating sites and clinical community*

Data collected may be shared in aggregate form in real time to inform the clinical community and facilitate discussion of clinical management of patients. These data will only be presented for all participants in total without identification of allocated treatments. Examples of such data will be baseline characteristics, investigation results, hospitalisation and ICU status, need for invasive or non-invasive ventilation, and mortality.

2.16 Dissemination policy

The trial results will be communicated to all site investigators by teleconference prior to publication or presentation. The trial results will also be submitted for presentation at national and international meetings and publications submitted to a peer reviewed scientific journal, irrespective of the results. A plain-language summary of the trial results will be made available to individual participants upon request.

Primary and senior authorship will be determined by the study steering committee. The authorship of the paper will include all of the Steering Committee who meet ICJME criteria

for authorship. Hospitals contributing at least one case for analysis will nominate a locally determined coordinating investigator for inclusion, in order of number of participants enrolled. All hospitals and participating organisations with protocols enacted will be listed as 'ASCOT group'. The ASCOT group will consist of all named site investigators and will be listed in the collaborators section of the paper. The author byline will include 'for the ASID Clinical Research Network'.

3. Appendices

These appendices will be updated as required during the study

3.1. Trial sites

See Appendix 1 (standalone document) for list of trial sites and site principal investigators.

3.2. Plans for biological specimens

There will be 4 tiers for collection of data and samples (see 2.5.6). The *Core* data only includes recording of clinical, outcome, treatment data and results from laboratory testing and does not involve storage of samples.

The *Enhanced biological, Research biological* and PK data and samples includes the collection and storage of some biological samples. The collection, processing, storage and shipping of biological samples will follow local standard operating procedures and regulations for handling and transporting clinical specimens containing infectious materials. These details will align with the Sentinel Travellers Research Preparedness Platform for Emerging Infectious Diseases (SETREP-ID) Biological Specimen Standard Operating Procedures.

These samples and data collection will be dependent on site capacity and participant consent obtained for enhanced sample collection, storage and sharing.

3.3. Trial Governance

3.3.1. Trial Steering Committee

The trial will be overseen by the Trial Steering Committee that will include:
Steven Tong, Justin Denholm, Joshua Davis, David Paterson, representatives from each participating jurisdiction, the ASID CRN, and a biostatistician.

3.3.2. Trial Management Committee

The trial management committee will oversee the day to day aspects of the trial. This committee will include:
Steven Tong, Justin Denholm, Joshua Davis, clinical trial manager and project officers.

3.3.3. Funding arrangements

Ongoing funding is being sought through philanthropy and applications to MRFF and the Victorian Medical Research Acceleration Fund.

3.4. Preparation of Study Drug Oral Solution

3.4.1 Hydroxychloroquine

Hazard: Do not crush the tablet if you are pregnant. Consider local handling precautions for hazardous medicines

The following information has been provided by the REMAP-CAP group and is in line with their current practises. The information applies to 200mg tablets.

3.4.1.1 For enteral feeding tubes:

1. Give immediately after a bolus feed or stop the continuous feed.
2. Flush the tube with 30 mL of water.
3. Crush the tablet to a fine powder using a mortar and pestle or a tablet crusher.
4. Add 10 mL of water to the powder and mix well (the tablet does not disperse easily).
5. Draw the mixture into the enteral syringe.
6. Rinse the crushing device with 10ml of water, then repeat with another 10ml (total of 20ml) and draw into the enteral syringe, to ensure that all of the medicine is removed.
7. Give the mixture (~30mL) immediately into the enteral feeding tube.
8. Rinse the enteral syringe with a further 10 mL of water to ensure the entire dose is given.
9. If other medicines are given, flush the tube with at least 5 mL of water between each medicine.
10. After the final medicine is given, flush the tube with 30 mL of water.
11. Restart the continuous feed immediately after dosing.

Smaller volumes can be used to accommodate fluid-restriction.

3.4.1.2 For swallowing difficulties:

1. Crush the tablet with a mortar and pestle or a tablet crusher.
2. Add 10 to 20 mL of water and mix well.
3. Draw the mixture into an oral dispenser/syringe.
4. Rinse the crushing device with 10ml of water, then repeat with another 10ml (total of 20ml) and draw into the oral dispenser/syringe, to ensure that all of the medicine is removed.
5. Give the mixture immediately.
6. Rinse the oral dispenser/syringe with a further 10mL of water and give to the patient, to ensure the entire dose is given.

If the person cannot swallow thin fluids (i.e. is an aspiration risk) or the very bitter taste is unacceptable, crush the tablet and mix with a spoonful of yoghurt or apple puree.

3.4.2 Lopinavir / Ritonavir

The Sponsor's first preference is to use commercially available oral suspension Lopinavir 80mg/Ritonavir 20mg per mL suspension. A limited number of lopinavir/ritonavir oral suspension stock has been secured from the National Medical Stockpile (Australian Government). Site access to this oral formulation is outlined in the IMP Management Plan. If during the study, the oral suspension is no longer available from the Sponsor, then we

recommend sourcing the oral solution from local pharmacy. If there is no access for local sites to secure commercially available oral solution, then the Sponsor recommends crushing the lopinavir/ritonavir study tablets. Sites are to contact the Sponsor for further guidance and information regarding the crushing of lopinavir/ritonavir tablets.

Swallowing difficulties:

1. Administer 5mL of suspension orally

The liquid has an unpleasant taste. The use of ice chips before and after the dose may numb the taste buds or mix the dose with chocolate syrup to mask the taste

Feeding tube:

1. Give immediately after a bolus feed or stop the continuous feed
2. Flush the tube with 30 mL of water
3. Draw 5mL into an enteral syringe and dilute with at least an equal volume of water
4. Mix well and give via the nasogastric feeding tube
5. A white residue or cloudy solution may be seen but will not clog the tube
6. Flush the tube with 30 mL of water and restart the feed

Due to the high content of propylene glycol and ethanol in the oral suspension, a PVC / silicone feeding tube should be used due to incompatibility with polyurethane.

There is adequate absorption of lopinavir when the oral liquid is given through nasogastric or PEG tubes and reduced absorption when given through jejunal tubes.

4. References

1. Guan WJ, Ni ZY, Hu Y, et al. Clinical Characteristics of Coronavirus Disease 2019 in China. N Engl J Med 2020.
2. Arabi YM, Alothman A, Balkhy HH, et al. Treatment of Middle East Respiratory Syndrome with a combination of lopinavir-ritonavir and interferon-beta1b (MIRACLE trial): study protocol for a randomized controlled trial. Trials 2018;19:81.
3. Chu CM, Cheng VC, Hung IF, et al. Role of lopinavir/ritonavir in the treatment of SARS: initial virological and clinical findings. Thorax 2004;59:252-6.
4. Chan KS, Lai ST, Chu CM, et al. Treatment of severe acute respiratory syndrome with lopinavir/ritonavir: a multicentre retrospective matched cohort study. Hong Kong Med J 2003;9:8.
5. Wang M, Cao R, Zhang L, et al. Remdesivir and chloroquine effectively inhibit the recently emerged novel coronavirus (2019-nCoV) in vitro. Cell Res 2020;30:269-71.
6. Sheahan TP, Sims AC, Leist SR, et al. Comparative therapeutic efficacy of remdesivir and combination lopinavir, ritonavir, and interferon beta against MERS-CoV. Nature communications 2020;11:222.
7. Cao B, Wang Y, Wen D, et al. A Trial of Lopinavir-Ritonavir in Adults Hospitalized with Severe Covid-19. N Engl J Med 2020.
8. Yao X, Ye F, Zhang M, et al. In Vitro Antiviral Activity and Projection of Optimized Dosing Design of Hydroxychloroquine for the Treatment of Severe Acute Respiratory Syndrome Coronavirus 2 (SARS-CoV-2). Clin Infect Dis 2020.
9. Taylor WRJ, Thriemer K, von Seidlein L, et al. Short-course primaquine for the radical cure of *Plasmodium vivax* malaria: a multicentre, randomised, placebo-controlled non-inferiority trial. Lancet 2019;394:929-38.
10. Russell CD, Millar JE, Baillie JK. Clinical evidence does not support corticosteroid treatment for 2019-nCoV lung injury. Lancet 2020;395:3.
11. Grasselli G, Pesenti A, Cecconi M. Critical Care Utilization for the COVID-19 Outbreak in Lombardy, Italy: Early Experience and Forecast During an Emergency Response. JAMA 2020.
12. International Conference on Harmonisation of technical requirements for registration of pharmaceuticals for human u. ICH harmonized tripartite guideline: Guideline for Good Clinical Practice. J Postgrad Med 2001;47:45-50.
